# Burden and sociodemographic determinants of pneumonia and diarrhoea among children younger than 5 years in Somalia: a community-based cross-sectional study

**DOI:** 10.1136/bmjopen-2024-098505

**Published:** 2025-11-09

**Authors:** Abdullahi Ahmed Tahlil, Mariam Muse Osman, Saido Gedi, Fartun Ali Haji, Abdulmunim Mohamed, Abdirashid Ali Asir Rage, Abdullah Al Azad, Iqbal Anwar, Abdirahman Abdirizak Ahmed, Yahye Sheikh Abdulle, Bashiru Garba, Mohamed Abdelrahman Mohamed

**Affiliations:** 1 Faculty of Medicine and Health Sciences, Zamzam University and Technology, Mogadishu, Somalia; 2 National Institute of Health, Ministry of Health and Human Services, Mogadishu, Somalia; 3 School of Public and Research, Somali National University, Mogadishu, Somalia; 4 Newborn and Child Unit, Ministry of Health and Human Services, Mogadishu, Somalia; 5 Department of Research, Training and Disease Surveillance, Benadir Maternity and Child Hospital, Mogadishu, Somalia; 6 South Health Campus, Alberta Health Services, Calgary, Alberta, Canada; 7 Department of Research and Planning, OGSB Hospital and Institute of Reproductive and Child Health, Dhaka, Bangladesh; 8 Faculty of Veterinary Medicine and Animal Husbandry, Somali National University, Mogadishu, Somalia; 9 Faculty of Medicine and Health, Jamhuriya University of Science and Technology, Mogadishu, Somalia; 10 SIMAD Institute for Global Health (SIGHt), SIMAD University, Mogadishu, Somalia; 11 Department of Public Health and Preventive Medicine, Faculty of Veterinary Medicine, Usmanu Danfodiyo University Sokoto, Sokoto, Nigeria

**Keywords:** Paediatric infectious disease & immunisation, Child, Cross-Sectional Studies, PUBLIC HEALTH

## Abstract

**Abstract:**

**Objective:**

Pneumonia and diarrhoea are two of the major causes of child mortality globally. Countries affected by conflict and other humanitarian emergencies, such as Somalia, have a particularly high burden of these diseases. Published reports from UNICEF and WHO have shown that various factors, including social, economic and environmental factors, are all associated with the occurrence of childhood pneumonia and diarrhoea. The objective of this study was to determine the prevalence, burden and associated sociodemographic determinants of pneumonia and diarrhoea among children younger than 5 years (under-5 children) in Somalia.

**Design:**

A community-based survey using an interviewer-administered questionnaire was conducted employing a modified WHO Expanded Program on Immunization (EPI) 30-Cluster sampling technique to identify households and respondents in nine selected districts across six member states in Somalia. The interviewers began selecting households starting from house number 1 and continued until 75 households were surveyed in each cluster.

**Setting:**

We considered the catchment areas of 12 target maternal and child health (MCH) centres as our study areas. Villages were considered as primary sampling units (PSU) while households within villages were considered as secondary sampling units, where women (with under-5 children) within households were the respondents.

**Participants:**

A total of 36 clusters (villages) were selected from the catchment areas of 12 target MCH centres. All households within the selected villages’ PSUs were listed. The interviewer started interviewing from house number 1 and continued till 75 households were covered to conduct interviews with mothers of under-5 children. Data collection took place between October and December 2023.

**Outcome measures:**

The prevalence and burden of childhood pneumonia and diarrhoea were estimated. A logistic regression model was employed to examine the determinants of childhood pneumonia and diarrhoea.

**Results:**

A total of 2483 under-5 morbidities were reported, 1712 probable pneumonia cases and 825 diarrhoea cases. Our calculations suggest that the prevalence of overall under-5 morbidity was 458.4 per 1000 children (95% CI 444.3 to 472.6) in the last 90 days. The prevalence of pneumonia and diarrhoea was 316.0 (95% CI 303.5 to 328.8) and 152.3 (95% CI 142.2 to 162.8) per 1000 under-5 children, respectively. A total of 70 under-5 deaths occurred in the past year, of which 37 were infants. Our exploration depicts an under-5 mortality rate of 39.3 deaths per 1000 live births per year (95% CI 30.6 to 49.7), and the infant mortality rate was 20.8 per 1000 live births per year (95% CI 14.8 to 28.6) in the study area, which is much lower than earlier estimates. The crude birth rate was 106.6 per 1000 population, and the stillbirth rate was 149.8 per 1000 births (95% CI 134.9 to 165.7), which is very high. We explored probable causes of 70 under-5 deaths and found that the highest proportion of under-5 deaths (22.9%) was due to acute respiratory infections (ARI), and about 15.7% were due to diarrhoea. Among other probable causes, congenital diseases (12.9%), accidents (11.4%) and measles (8.6%) were noteworthy.

**Conclusion:**

This study revealed a high burden of pneumonia and diarrhoea among the studied population in Somalia. The study also identified important sociodemographic and environmental determinants that tend to increase the risk of pneumonia and diarrhoea among under-5 children.

**STRENGTHS AND LIMITATIONS OF THIS STUDY**

- This study uses a modified WHO Expanded Program on Immunization (EPI) Cluster sampling technique, which enhances the representativeness of the sample by capturing diverse households within selected villages, thereby improving the validity of the findings.
- This study uses advanced statistical methods such as multivariate logistic regression and principal component analysis to analyse and interpret complex relationships between variables, improving the depth of analysis regarding socioeconomic inequities.
- This study uses a cross-sectional design, which limits the ability to capture temporal associations or seasonal variations in the incidence of pneumonia and diarrhoea among under-5 children, particularly given the higher prevalence of these illnesses during the wet season.
- This study did not assess breastfeeding practices as a potential sociodemographic determinant, which might have provided valuable insights into the relationship between infant feeding practices and the prevalence of pneumonia and diarrhoea in the target population.

## Introduction

 Pneumonia and diarrhoea are the leading causes of childhood morbidity and mortality in low- and middle-income countries, including Somalia.^1^,^4^ They are responsible for approximately 2.3 million deaths among children younger than 5 years (under-5 children) globally each year, accounting for 29% of all childhood deaths.^5^ Despite significant declines in child mortality between 2000 and 2010, diarrhoea and pneumonia remain the leading causes of preventable deaths, accounting for approximately 30% of all child fatalities worldwide. Therefore, the attainment of the fourth Millennium Development Goal and the long-term objective of reducing child mortality to 20 deaths or fewer per 1000 live births in all countries by 2035 will require substantial decreases in mortality from these two diseases.^6^

In Africa, the prevalence of pneumonia and diarrhoea is a significant public health concern, with an estimated 45% of all deaths among under-5 children attributed to these conditions.^6^ In sub-Saharan Africa, the mortality rates for children are high, with a mortality rate of 74 deaths per 1000 live births, which is a staggering 14 times higher than the mortality rates observed in Europe and North America.^7^ In East Africa, the prevalence of pneumonia and diarrhoea was found to be particularly high, with rates of 34% and 14.3%, respectively.^8 9^

In Somalia, the under-5 mortality rate (U5MR) is among the highest globally, with an estimated 117 deaths per 1000 live births.^10^ This rate is higher than the sub-Saharan African average of 76 deaths per 1000 live births and significantly higher than the global average of 36 deaths per 1000 live births.^11^ Notably, pneumonia and diarrhoea are major contributors to these alarming mortality rates, accounting for approximately 24% for pneumonia and 19% for diarrhoeal diseases, respectively.^12^ This substantial burden of disease can be attributed to a multifaceted range of factors, including limited access to healthcare services, poor sanitation and hygiene practices, and inadequate nutrition.

Notably, Somalia ranks as the second-highest country worldwide in terms of pneumonia burden and mortality, with an estimated 15 165 under-5 children succumbing to pneumonia in 2019, representing approximately 21% of all child deaths.^13 14^ This statistic underscores the severity of the situation, indicating that at least two children die every hour due to pneumonia in the country.^15^ The burden of under-5 morbidity and mortality in Somalia is also high compared with neighbouring African and Asian countries.^14^

Despite global efforts to improve child health and reduce childhood mortality, pneumonia and diarrhoea remain the leading causes of morbidity and mortality among under-5 children in Somalia, with significant consequences for child health, development and socioeconomic well-being. However, there is limited context-specific evidence on the sociodemographic determinants and burden of these diseases within different regions of Somalia, which hampers the development of targeted interventions. The high prevalence of these diseases in Somalia is a major public health concern, particularly amid ongoing humanitarian crises and limited access to healthcare services. The lack of vaccination is also a key factor, addressing the broader issue of immunisation and its impact on the health of under-5 children in Somalia. Therefore, this study aims to determine the burden and identify key sociodemographic determinants of pneumonia and diarrhoea among under-5 children in Somalia’s humanitarian settings. This study contributes to the existing literature by specifically examining the burden and sociodemographic determinants of these diseases within the Somali context, thereby filling a critical gap in localised data. It aims to fill critical gaps in understanding how various determinants influence disease prevalence, thereby informing more targeted and effective public health interventions. Ultimately, the findings are expected to support policymakers and healthcare providers in designing tailored strategies to reduce disease burden and improve child health outcomes in this vulnerable population.

## Methods

### Study design and setting

A community-based cross-sectional study was conducted over a period of 18 months in nine districts of Somalia (online supplemental table 1). These districts were targeted for this project based on having high numbers of under-5 children, having the highest burden of child pneumonia and diarrhoeal diseases among under-5 children, being severely affected by the drought with a large number of people displaced and, as at the time of the study, not supported by any other agencies for primary healthcare (PHC) services.

**Figure 1:**
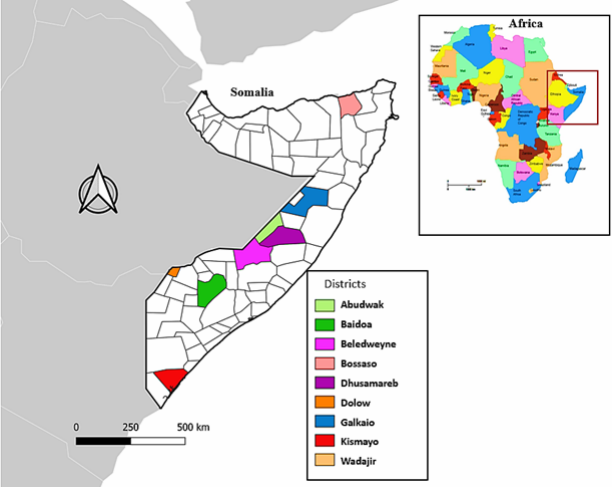
Geographical areas of the study showing the nine selected districts in Somalia.

Of the 2.8 million people estimated to be living in these nine districts, approximately 827 322 are affected by the severe drought of 2022/2023, which swept across the country as shown in online supplemental table 1. The study was conducted in 12 PHC/maternal and child health (MCH) clinics, which act as referral hospitals for the nine districts. The location of these nine district hospitals (referral) and 12 health facilities (PHC/MCH centres) is shown in figure 1.

### Sampling

For the community survey, we followed a modified WHO Expanded Program on Immunization (EPI) Cluster sampling technique to identify households and respondents in the community to conduct interviews. We considered the catchment areas of 12 target MCH centres as our study areas. Villages within these catchment areas were selected as primary sampling units (PSUs). The villages were chosen using a systematic random sampling method from the list of all villages in each catchment area to ensure representativeness. A total of 36 villages (clusters) were selected from the combined catchment areas of the 12 target MCH centres. All households within these selected villages were listed, and women with under-5 children within these households were identified as respondents. The interviewer started interviewing from house number 1 and continued till 75 households were covered to conduct interviews with mothers of children under 5 years of age. If more samples were required to cover 75 mothers with under-5 children in a village, the interviewer included households from the adjacent village to cover the interview target. If there were more than one under-5 child in a household, the mother of the youngest child was selected as the respondent. Households without any under-5 children were excluded systematically.

### Sample size estimation

For estimating the minimum sample size, we used the existing prevalence rate of 3 outcome indicators: (1) care seeking for pneumonia, (2) prevalence of pneumonia and diarrhoea and (3) U5MR (table 1). Taking 1.55 as the design effect, since we deviated from simple random sampling, we needed 1785×1.55=2767 households to visit. It implies that following the EPI 36 cluster sampling methodology, respondents needed to visit at least 75 households in a village to interview mothers of under-5 children.

**Table 1:** Sample size calculation

Indicator	Current prevalence	Expected rate after 1 year of intervention	Power	Level of confidence	Ratio	Minimum sample req. in each survey
Under-5 mortality rate	117/1000 live-births	88/1000 live-births	80%	95%	1	1785
Prevalence of under-5 pneumonia per 100 in the last 90 days	50%	40%	80%	95%	1	408
Visit a health facility for the treatment of pneumonia	13%	26%	80%	95%	1	160

### Data collection tools and procedures

A semistructured questionnaire (online supplemental file) was administered face-to-face by trained data collectors, with closed, semi-closed and open-ended questions to address the study objectives. The questionnaire was developed in English but translated into Somali before administration. Before administration, the questionnaire was back-translated to ascertain consistency. Behaviour and quality of service were also assessed using client-reported Likert-scale items adapted from the WHO standard patient satisfaction survey modules. Respondents were asked to rate the behaviour of service providers as ‘excellent’, ‘good’, ‘satisfactory’, ‘bad’ or ‘very bad’, and the overall quality of care as ‘excellent’, ‘good’, ‘satisfactory’ or ‘poor’, based on their most recent visit for treatment of pneumonia or diarrhoea.

The field-level data collection was organised by the Somali National Institute of Health and the Ministry of Health, with nominated team members. 11 data collectors were deployed to collect data from the field. Two supervisors and a team leader were assigned to support field data collection, maintaining high data quality. All of the data collectors were trained for 3 days on data collection tools and techniques. After the 2nd day of training, the enumerators were sent to a hypothetical field (outside the study area) to pretest the tool. The pretest experience was reviewed together with the enumerators and their supervisors, and necessary changes were made to finalise the tool to facilitate quality data collection during the actual survey. All data were collected online using a tablet/smartphone. Field data collection took place between October and December 2023.

### Data management and analysis

After completion of field data collection, the database was transferred to Microsoft Excel and reviewed daily by supervisors and study investigators for quality assurance. Any inconsistencies were flagged and communicated to the data collectors for correction, which included revisiting households when necessary. Once the data were verified and cleaned, the final dataset was transferred to SPSS V.24 for comprehensive statistical analysis.

To account for the complex survey design, particularly the use of a modified WHO EPI 30-Cluster sampling approach, sampling weights were computed and applied in the analysis. The weights were calculated as the inverse of the probability of selection at each stage of the sampling process. Specifically, this considers both the probability of selecting a given village (PSU) within a district and the probability of selecting a household within that village. Although a fixed number of households (75) were interviewed per village, the underlying populations of districts and clusters varied, which could introduce selection bias if not adjusted for. Therefore, these sampling weights ensured that the survey results more accurately reflected the underlying population structure across the nine districts and six member states included in the study. The weighted dataset was then used to produce both descriptive and inferential statistics.

Frequency distributions of all relevant outcome indicators (eg, under-5 morbidity and mortality, care-seeking behaviour) were generated. Sociodemographic characteristics were summarised and presented using both absolute numbers and corresponding percentages for clarity and ease of interpretation, as recommended in reporting guidelines for descriptive epidemiology. For assessing associations between sociodemographic variables and child health outcomes, multivariate logistic regression models were developed. The results of these models were presented as crude and adjusted ORs (AORs) with 95% CIs.

Additionally, a wealth index was constructed using principal component analysis (PCA) and factor analysis (FA) to categorise households into quintiles based on asset ownership and housing characteristics. These included household possessions (eg, refrigerator, television), type of latrine, source of drinking water, construction material of the dwelling and ownership of farming land or livestock. Household income and maternal education were analysed separately and were not included in the asset-based wealth index.

Multivariate logistic regression models were developed for each of the three key outcome variables: (1) any under-5 morbidity, (2) pneumonia within the last 90 days and (3) diarrhoea within the last 90 days. Covariates included in the models were selected based on theoretical relevance and evidence from previous literature. These included asset-based wealth quintile (constructed via PCA and FA), maternal education, maternal occupation, region, area of residence (urban/rural/Internally Displaced Person), type of cooking material (gas/electricity vs charcoal/wood), source of drinking water (safe vs unsafe) and type of latrine used by the household (sanitary vs unsanitary). ORs (both crude and adjusted) with 95% CIs were reported for each variable to determine the strength and direction of associations with the outcome variables.

All collected data were reviewed daily, and households with identified anomalies were revisited. After the quality control process, missing data were minimised to less than 1% of the total data set, thereby ensuring the overall quality and completeness of the data.

### Patient and public involvement

This study was designed with input from the local communities in Somalia, specifically focusing on the perspectives and experiences of caregivers and families with under-5 children affected by pneumonia and diarrhoea. Community representatives were engaged during the planning phase to ensure that the research objectives aligned with the needs and priorities of the population. Patients and caregivers were not directly involved in the recruitment and conduct of the study; however, their feedback was instrumental in shaping the research questions and identifying key sociodemographic factors pertinent to the burden of these illnesses. Discussions with community leaders and health workers provided valuable insights into the challenges faced by families, which guided the development of our study methodologies. The findings of this study will be disseminated to the communities from which participants were drawn, ensuring that the results are communicated in a culturally appropriate and accessible manner. We also aim to involve community stakeholders in the discussion of the findings and in the development of strategies to address the burden of pneumonia and diarrhoea among under-5 children in the context of fragile humanitarian settings. This collaborative approach is intended to foster a sense of ownership among the community and enhance the translation of research findings into practice.

## Results

### Sociodemographic characteristics

A total of 2767 households were approached, of which 2712 households participated in the study, resulting in a response rate of 98%. A total of 55 eligible households declined to participate in the study. One woman from each participating household (the mother of the under-5 child) was interviewed to address the study objectives across 36 villages within the catchment areas of 12 MCH centres. Households without under-5 children were excluded. Table 2 shows that the mean age of respondents was 29.4±7.07 years, with the majority, 1162 (42.8%), aged between 20 and 29 years. Most respondents had an Islamic education 1341 (49.4%), and the vast majority were housewives 2134 (78.7%) by occupation. Of the 2712 households, 2193 (80.9%) were from urban areas. The regional distribution shows the highest proportions in Banadir 551 (20.3%) and Bay 540 (19.9%). The average household size was 6.25±2.9 members per household, with over half (56.3%) having seven or fewer members. The median monthly household income was US$180, with 2372 (87.5%) earning less than US$500 per month, highlighting the prevalence of low-income households. Regarding reproductive characteristics, most respondents 2457 (90.6%) were multiparous, with an average parity of 4.7±4.0. Only 777 (28.7%) of households had sanitary latrines, and their primary drinking water source was mainly piped water 2123 (78.3%). The construction materials and the number of bedrooms in the main dwelling indicate a much higher prevalence of poor and middle-class households (table 2).

**Table 2:** Sociodemographic characteristics of respondents (N=2712)

Indicator	Status	Frequency	Percentage
Age (in years)	<19	316	11.7
	20–29	1162	42.8
	30–39	965	35.6
	40–49	269	9.9
Education of respondents	No education	648	23.9
	Islamic	1341	49.4
	Primary	403	14.9
	Secondary dropout	125	4.6
	Secondary	99	3.7
	Higher secondary and above	96	3.5
Occupation of respondents	Business	199	7.3
	Day-labour	205	7.6
	Housewife	2134	78.7
	Service (private/public)	174	6.4
Settlement type	IDP	336	12.4
	Rural	183	6.7
	Urban	2193	80.9
Region	Banadir	551	20.3
	Bari	360	13.3
	Bay	540	19.9
	Galgadud	358	13.2
	Gedo	360	13.3
	Hiran	180	6.6
	Lower Jubba	181	6.7
	Mudug	182	6.7
Household size	≤7	1526	56.3
	>7	1186	43.7
Household monthly income in USD	<500	2372	87.5
	≥500	340	12.5
Parity of respondents	Primiparous	255	9.4
	Multiparous	2457	90.6
Construction material of the roof	Concrete	444	16.4
	Tin	2129	78.5
	Plastic sheet	62	2.3
	Grass/leaves	77	2.8
Construction material wall	Concrete	970	35.8
	Tin	1382	51.0
	Mud	157	5.8
	Grass/leaves	183	6.7
	Others	20	0.7
Construction material of the floor	Concrete	1163	42.9
	Wooden	264	9.7
	Mud	1274	47.0
	Others	11	0.4
Type of latrine used by household members	Sanitary	777	28.7
	Water-seal	1405	51.8
	Open space	467	17.2
	Others	63	2.3
Source of drinking water	Piped water	2123	78.3
	Tube-well	449	16.6
	Surface water	140	5.2
Number of bedrooms	1	685	25.3
	2	1052	38.8
	3	579	21.3
	4	255	9.4
	≥5	141	5.2
Cooking materials	Gas and electricity	453	16.7
	Charcoal and wood	2259	83.3

IDP, Internally Displaced Person.

**Table 3:** Population, births and under-5 and infant death rates

Indicator	Number/proportion
(A) Total no. of households	2712
(B) Total population covered	19 660
(C) Total no. of births in the last 1 year	2096
(D) Total no. of live births in the last 1 year	1782
(E) Total no. of stillbirths in the last 1 year	314
(F) Total under-5 deaths in the last 1 year	70
(G) Total no infant deaths in the last 1 year	37
(H) Total under-5 population	5417
(I) Crude birth rate ((C/B) × 1000)	106.6 births per 1000 population per year
(J) Under-5 mortality rate ((F/D) × 1000)	39.3 deaths per 1000 live births per year
(K) Infant mortality rate ((G/D) × 1000)	20.8 per 1000 live births per year
(L) Stillbirth rate ((E/C) × 1000)	149.8 per 1000 births
(M) Proportion of under-5 population (H/B) × 100	27.6%

The possessions of the households demonstrate that a small proportion of households have amenities like freezer (8.1%), microwave (0.5%), radio (5.6%), computer (6.9%), air-conditioner (2.0%), motorcycle (1.9%) or bicycle (0.8%), but many own ceiling fans (30.3%) and televisions (24.8%). About 15% of households own livestock, and 7.7% of households own farming lands. 92.3% of households own mobile phones, and the majority (78.6%) have mobile accounts as opposed to 9.8% who have real bank accounts. 7.4% of the households have Wi-Fi connection in their houses, and 42.8% households access the internet through mobile data packages.

### Under-5 mortality and its causes

Among 2712 households surveyed, a total of 19 660 individuals were residing, of whom 5417 were under-5 children (27.6%). During the last year (before the data collection date), a total of 2096 births took place in these households, of which 1782 were live births and 314 were stillbirths. During the last year, a total of 70 under-5 deaths took place, of which 37 deaths took place at an age below 1 year (infants). Our exploration depicts an U5MR of 39.3 deaths per 1000 live births per year (95% CI 30.6 to 49.7) and an infant mortality rate of 20.8 per 1000 live births per year (95% CI 14.8 to 28.6) in the study area, which is much lower than earlier estimates. The crude birth rate in the study area is 106.6 births per 1000 population, while the stillbirth rate is 149.8 per 1000 births (95% CI 134.9 to 165.7), which is very high. (table 3)

**Figure 2:**
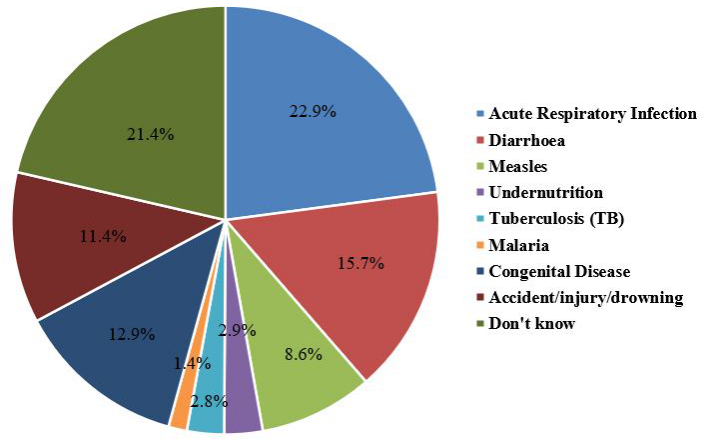
Probable cause of under-5 deaths during the last year.

We explored probable causes of 70 under-5 deaths and found that the highest proportion of under-5 deaths (22.9%) was due to acute respiratory infections (ARIs), and about 15.7% were due to diarrhoea. Among other probable causes, congenital diseases (12.9%), accidents (11.4%) and measles (8.6%) were noteworthy (figure 2).

### Under-5 morbidity

We asked a number of questions in the survey to estimate the overall prevalence of childhood morbidity and morbidities from pneumonia and diarrhoea during the last 90 days prior to the survey date. A total of 2483 under-5 morbidities were reported, of which 1712 were probable pneumonia cases and 825 were diarrhoea cases. Our calculations suggest that the prevalence of overall under-5 morbidity was 458.4 per 1000 children (95% CI 444.3 to 472.6) in the last 90 days. The prevalence of pneumonia and diarrhoea was 316.0 (95% CI 303.5 to 328.8) and 152.3 (95% CI 142.2 to 162.8) cases per 1000 children, respectively. In another way, 63.1% of families had at least one pneumonia case, and 30.4% had at least one diarrhoea case within the last 90 days. The burden of pneumonia was higher than that of diarrhoea but comparable with earlier estimates (table 4).

**Table 4:** Total childhood morbidity and morbidities from pneumonia and diarrhoea

Indicator	Measurement
(A) Total no. of households visited	2712
(B) Total no. of under-5 children	5417
(C) Total no. of under-5 morbidity in the last 90 days	2483
(D) Total no. of under-5 pneumonia in the last 90 days	1712
(E) Total no. of under-5 diarrhoea in the last 90 days	825
(F) Prevalence of overall under-5 morbidity (per 1000 children per 3 months) ((C/B) × 1000)	458.4
(G) Prevalence of pneumonia/ARI among U5 children (per 1000 children per 3 months) ((D/B) × 1000)	316.0
(H) Prevalence of diarrhoea among U5 children (per 1000 children per 3 months) ((E/B) × 1000)	152.3
(I) % of households having at least one pneumonia case in the last 90 days ((D/A) × 100)	63.1%
(J) % of households having at least one diarrhoea case in the last 90 days ((E/A) × 100)	30.4%

ARI, acute respiratory infection.

### Care-seeking practices for pneumonia, diarrhoea and for conducting the last childbirth

**Figure 3:**
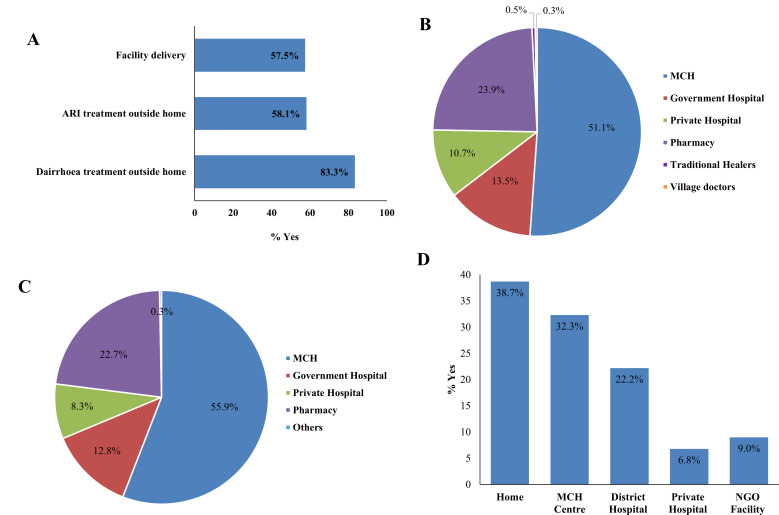
(A) Care seeking outside home for under-5 pneumonia, diarrhoea and conducting deliveries; (B) place of treatment for under-5 pneumonia cases; (C) place of treatment for diarrhoea of under-5 children; (D) place of last birth within the family. ARI, acute respiratory infection; MCH, maternal and child health; NGO, non-governmental organisation.

We explored care seeking for the last pneumonia and diarrhoea cases that took place in the surveyed households within the last 90 days. Also, we investigated where the last birth took place and who conducted that birth. The result suggests that those under-5 children who suffered from pneumonia, among them 58.1% went outside home for treatment, while the same proportion for treatment of childhood diarrhoea was 83.3%. The facility delivery rate in the study area was 57.5% (figure 3A). Among those who went outside their homes for treatment of pneumonia, 51.1% went to MCH centres, 13.5% to government hospitals and 10.7% went to private hospitals. It is surprising to note that about 23.9% of pneumonia cases visited pharmacies for treatment (figure 3B). A similar pattern of health facility use was observed for treatment of under-5 diarrhoea, where 55.9% visited MCH centres, 22.7% sought care in pharmacies and 12.8% went to government hospitals for treatment of diarrhoea (figure 3C). Of all births, 37.8% took place at home, 32.3% in MCH centres and 22.2% in government hospitals (district hospitals). The contribution of the private or non-governmental organisation sector was minimal in Somalia for maternal and child healthcare (figure 3D).

### Distance, travel time, cost and perceived quality of care in health facilities

To access care for pneumonia from health facilities, household members had to cover a mean distance of 2.94 km, which required, on average, 20.6 min; while children with diarrhoea had to cover almost the same distance, which required 17 min of time on average. The average cost of treatment for one episode of pneumonia was US$13, while the same was US$8.5 for the treatment of diarrhoea.

### Quality of care

**Figure 4:**
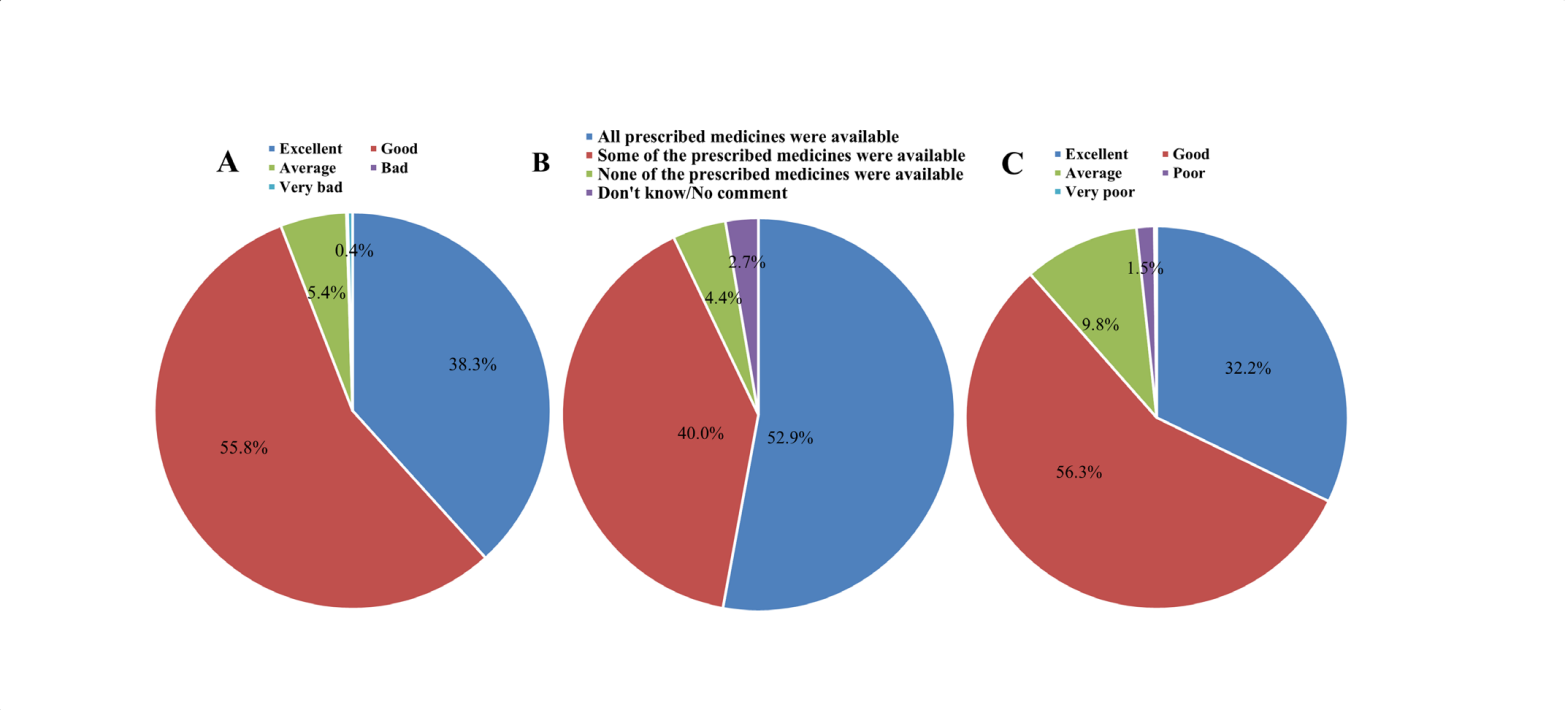
(A) Behaviour of service providers during last visit for care of children; (B) Availability of medicines during last facility visit; (C) Overall quality of care in health facilities as perceived by users.

When asked about the behaviour of service providers during the last facility visit for treatment of pneumonia or diarrhoea, 35.3% mentioned that the behaviour was ‘excellent’ and 55.8% mentioned behaviour as ‘good’ (figure 4A). The behaviour of service providers was better in public sector facilities than in private sector facilities (data not shown). Data suggests that Somali people are very satisfied with whatever care they receive from health facilities. The overall quality of care in the healthcare facility was mostly good (53%) as perceived by the users, and a significant number of users (32%) perceived the quality of care to be excellent (figure 4B).

About the availability of medicines, 52.9% respondents mentioned that all prescribed medicines were available from health facilities, and another 40% mentioned that some of the prescribed medicines were available, and only 2.6% mentioned that none of the prescribed medicines were available from the health facility visited for treatment of diarrhoea or pneumonia (figure 4C). When asked about treatments received for pneumonia, the majority of the respondents mentioned being prescribed an antibiotic tablet (72%); following this, 35% children received syrup or injection (bronchodilator), and 34% received an antibiotic injection. But the minimum number of children suffering from ARI who received oxygen and nebuliser, only 11% received oxygen and 4.6% received nebuliser while receiving treatment from healthcare facilities. From the medicines prescribed for diarrhoea, most of the children received a zinc tablet or syrup (87%) and oral rehydration solutions (ORS) (85.6%). Apart from this, more than 43% of children suffering from diarrhoea received intravenous fluids, and almost 27% of children were treated with antibiotics.

### Performance of community health workers

We explored the performance of the existing community health workers (CHWs) in terms of the number of home visits made by them during the last 3 months and the services provided during their last home visit. Only 37.2% households received at least one visit by CHWs, and the mean number of visits made by the CHWs in the last 3 months was 1.8, and the median number of visits made by the CHWs was 2 in the last 3 months. Several preventive and promotive healthcare services were provided during the last home visit of the CHWs. Among the services provided by the CHWs, the most frequent one was health education (87.6%), but provision of other essential services was low during their last home visit, such as home management of pneumonia 6%, management of diarrhoea 8.5%, antenatal care (ANC) 12% and postnatal care (PNC) 3.4%.

### Sociodemographic determinants for outcome indicators

To identify the key sociodemographic determinants of childhood pneumonia, diarrhoea and overall morbidity, we developed multivariate logistic regression models using the cleaned and weighted data set. The primary outcome variables were (1) any under-5 morbidity in the last 90 days, (2) pneumonia in the last 90 days and (3) diarrhoea in the last 90 days. All models controlled for a consistent set of independent variables: household asset quintile, maternal education, maternal occupation, type of cooking material, type of latrine, source of drinking water, region and settlement type. These variables were included based on their plausible influence on child health and findings from previous studies in similar settings.

Table 4 shows that 71% families had at least one U5 morbidity within the last 90 days. The prevalence varied significantly by socioeconomic status (SES), maternal education, occupation of mother, cooking materials, source of drinking water and region. A child from a household in the richest quintile had significantly decreased odds of having any under-5 morbidity (AOR 0.30; 95% CI 0.20 to 0.45) than a child from the poorest quintile household after adjusting for all covariates in the model. Similarly, overall disease prevalence was 87.8% for children of illiterate mothers, and the same rate was 56.4% among children with maternal education of secondary or above. A child of a mother with secondary education had significantly decreased odds of suffering from any childhood morbidity (AOR 0.51; 95% CI 0.32 to 0.80) than a child of an illiterate mother. Overall, under-5 morbidity also varied by occupation of the mother; children of service women had significantly decreased odds of suffering from morbidities compared with children of housewives. Morbidity also varied by region, cooking materials used and source of drinking water, but not by settlement type. Households using charcoal and wood as cooking materials had about twice the odds of having childhood morbidity (AOR 1.82; 95% CI 1.32 to 2.51) than households using gas or electricity for cooking.

About 47.4% of families had morbidity from pneumonia in the last 90 days, which varied significantly with SES, maternal education, occupation of mother, area and region after adjusting for all covariates in the model. Children from the richest quartile households had significantly decreased odds of having pneumonia (AOR 0.52; 95% CI 0.36 to 0.75), and children of mothers with secondary and above education also had significantly decreased odds of having pneumonia in the last 3 months (AOR 0.49; 95% CI 0.33 to 0.75). A similar result was found for children of service women (AOR 0.49; 95% CI 0.33 to 0.74). Prevalence of pneumonia also significantly varied by cooking materials (AOR 1.77; 95% CI 1.30 to 2.39), settlement type, region and source of drinking water. In the case of under-5 diarrhoea in the last 3 months, the overall prevalence was 40.3%, which varied significantly with SES and area of residence but not with maternal education, occupation of mother or region. Children from the richest quintile had lower odds of experiencing diarrhoea (AOR 0.67; 95% CI 0.40 to 1.13) compared with those from the poorest quartile. However, this association was not statistically significant. Children belonging to the richest quintile had decreased odds of having diarrhoea (AOR 0.67; 95% CI 0.40 to 1.13) compared with the poorest quintile; however, this difference was not statistically significant. The prevalence of diarrhoea among children of housewives was 42.3% and this prevalence was 53.1% among under-5 children who were displaced. Children from households with sanitary latrines had a prevalence of under-5 diarrhoea of 38.3%, whereas children from households without sanitary latrines had a 48.7% prevalence of under-5 diarrhoea. Households that have access to safe drinking water had a 40.1% prevalence of under-5 diarrhoea, but for households that do not have access to safe drinking water, this prevalence is 45.2% (online supplemental table 2).

Online supplemental table 3 demonstrates that the overall use of health facilities for the treatment of pneumonia varied significantly by region. The overall use of health facilities for the treatment of pneumonia was 53.5%. The highest use of health facilities for the treatment of pneumonia was reported from Gedo (57.7%) region, while the lowest use rate was found in Mudug region (42.9%). 60.6% children from the richest quintile households used health facilities for treatment of pneumonia compared with a 54.3% use rate among the children from the poorest quintile households. Children from the richest quintile households had increased odds of visiting health facilities for treatment (OR 1.67; 95% CI 0.96 to 2.94) compared with children from the poorest households. Nonetheless, this association was not statistically significant. Among the children of service women, 61.4% used care from a health facility, compared with 53.8% of children of the housewives. The overall use of health facilities for the treatment of diarrhoea was 84.9%, which varied significantly by maternal education, area of residence and region after adjusting for all covariates in the model. The overall use of health facilities for the last delivery was 62.2%. This use varied significantly by SES and maternal education, but not by occupation of mother, area of residence or region, after adjusting for all covariates. A child from a household in the richest quartile had significantly increased odds of being delivered in a health facility than a child from the poorest quartile (AOR 4.25; 95% CI 2.80 to 6.46). Children of mothers with secondary and above education were also significantly more likely to be delivered in a health facility than children of illiterate mothers (AOR 3.62; 95% CI 2.11 to 6.20).

## Discussion

This study aimed to determine the burden and sociodemographic determinants of pneumonia and diarrhoea among under-5 children in Somalia. We found a high prevalence of both conditions, with pneumonia more common than diarrhoea. The analysis also identified maternal education, household wealth, cooking practices and access to safe water as significant predictors of child morbidity and healthcare access.

The findings revealed a high burden of childhood morbidity with 316 cases of pneumonia and 152 cases of diarrhoea per 1000 under-5 children within the previous 90 days, resulting in an overall morbidity rate of 458 per 1000. The U5MR was 39.3 per 1000 live births, and the infant mortality rate was 20.8 per 1000 live births. The U5MR observed in this study (39.3 per 1000 live births) is notably lower than previously reported national figures. This finding is consistent with a previous study in Kenya and Ethiopia, which found U5MRs of 41 and 52 per 1000 live births.^16 17^ However, it is important to note that this study is not nationally representative, as it focused on catchment areas of targeted MCH centres. Still, the proportion of deaths attributed to pneumonia (22.9%) and diarrhoea (15.7%) is higher than global estimates. According to UNICEF, pneumonia accounts for about 13% and diarrhoea for 9% of under-5 deaths globally.^18^ Our findings underscore the amplified risks in conflict-affected populations, consistent with prior studies in emergency or refugee settings where pneumonia and diarrhoea contribute to 20% and 7% of child deaths, respectively.^3^

The findings confirm that morbidity and mortality from pneumonia and diarrhoea are exacerbated by poor access to clean water, inadequate sanitation, malnutrition and displacement, all prevalent in Somalia. In conflict-affected or fragile regions, weakened health systems and displacement worsen children’s vulnerability. Diseases that are otherwise preventable and treatable become life-threatening. This reinforces the urgency of addressing basic public health needs in such contexts. This study also explored healthcare-seeking behaviour and the performance of CHWs. Among children with pneumonia, 58.1% sought care, and among those with diarrhoea, 83.3% sought care. However, nearly a quarter of both groups relied on pharmacies rather than formal healthcare facilities. This mirrors findings from studies in Dhaka, Bangladesh, where pharmacies are often preferred due to their accessibility, immediate service and lack of consultation fees.^19^ These findings raise concerns about the quality and appropriateness of care received, as unregulated treatment from pharmacies can contribute to antimicrobial resistance and poor outcomes.

On the positive side, the perceived quality of care among users was generally high. Most caregivers rated their experience as good (53%) or excellent (32%). Such positive perceptions are important for promoting continued utilisation of healthcare services. However, the study found limited availability of essential treatments like oxygen or nebulisation for pneumonia, highlighting resource gaps in facilities. Only 11% of pneumonia cases received oxygen, and just 4.6% received nebuliser therapy, despite these being essential components of effective treatment. The study also assessed CHWs’ involvement. Only 37.2% of households had received a CHW visit in the previous 3 months. Most visits focused on health education (87.6%), but few involved clinical management of childhood illnesses—only 6% managed pneumonia and 8.5% diarrhoea. These findings suggest an underused opportunity to strengthen community-based healthcare delivery, particularly in hard-to-reach areas.

Sociodemographic analysis revealed sharp inequities in health outcomes. Children in the poorest quintile had significantly increased odds of suffering from pneumonia and diarrhoea compared with those in the richest quintile. Children from wealthy households had significantly decreased odds of having any morbidity (AOR 0.30; 95% CI 0.20 to 0.45) and significantly decreased odds of having pneumonia (AOR 0.52; 95% CI 0.36 to 0.75). However, while children from the wealthiest households had decreased odds of having diarrhoea (AOR 0.67; 95% CI 0.40 to 1.13), this association was not statistically significant. Maternal education was also protective: children of mothers with secondary education or higher had significantly decreased odds of suffering from pneumonia (AOR 0.49; 95% CI 0.33 to 0.75) and from any morbidity (AOR 0.51; 95% CI 0.32 to 0.80) than those of illiterate mothers. These findings are consistent with studies in West Africa and Ethiopia, where maternal literacy and income status significantly reduce disease burden.^20^,^22^ Moreover, maternal employment was associated with lower morbidity—children of service-working mothers had decreased odds of being ill compared with those of housewives. This finding is consistent with previous studies where a lack of employment was found to be significantly associated with childhood pneumonia and diarrhoea.^21^

Furthermore, the use of health facilities for childbirth was significantly higher among the richest households (OR 4.25; 95% CI 2.80 to 6.46) and among educated mothers (OR 3.62; 95% CI 2.11 to 6.20), which reflects existing structural inequities in access to quality maternal healthcare. Household environmental factors also influenced outcomes. Use of charcoal and wood for cooking significantly increased the risk of pneumonia (OR 1.77; CI 1.30 to 2.39), consistent with a study in Gamo Zone, Southern Ethiopia, where the place of food cooking was found to be significantly associated with pneumonia among under-5 children.^22^ Similarly, households without access to sanitary latrines or safe water had higher diarrhoea prevalence, reinforcing the role of Water, Sanitation and Hygiene (WASH) interventions in disease prevention.

In this study, we interpret associations as statistically significant only when the 95% CI does not include 1.0 and the p value is less than 0.05. Terms such as ‘more likely’ or ‘less likely’ are used without qualifiers only when results meet these criteria. When statistical significance is not achieved, we explicitly state that the observed differences are ‘not statistically significant’ to ensure accurate and transparent interpretation of findings.

Despite the comprehensive findings, this study has several limitations. First, this study uses a cross-sectional design, which limits the ability to establish causal relationships between exposures and health outcomes and does not capture temporal associations or seasonal variations in the incidence of pneumonia and diarrhoea among under-5 children, particularly given the higher prevalence of these illnesses during the wet season. Additionally, reliance on caregivers’ recall for morbidity data over the preceding 90 days may introduce recall bias, potentially leading to underestimation or overestimation of true prevalence. The study areas were selected based on 12 MCH catchment areas, which may limit the generalisability of the findings to the entire Somali population. Furthermore, the study did not assess breastfeeding practices or collect anthropometric indicators of child nutritional status, both of which are known to influence childhood immunity and vulnerability to infections such as pneumonia and diarrhoea. Finally, potential confounding effects of these unmeasured nutritional factors could not be adjusted for in the analysis. Despite these limitations, the large sample size and rigorous analysis strengthen the validity of our findings.

## Conclusion

This study revealed a high burden of pneumonia and diarrhoea among the studied population in Somalia. The study also identified important socioeconomic and environmental determinants that tend to increase the risk of pneumonia and diarrhoea among under-5 children. The logistic regression model found that maternal education, maternal employment status, source of drinking water and household income are all significant contributors to morbidity.

## Supplementary material

10.1136/bmjopen-2024-098505 online supplemental file 1

10.1136/bmjopen-2024-098505 online supplemental file 2

## Data Availability

Data are available upon reasonable request.
