# Supplementary material for: Burden and sociodemographic determinants of pneumonia and diarrhoea among children younger than 5 years in Somalia: a community-based cross-sectional study
Source: BMJ Open. 2025 Nov 9;15(11):e098505. doi: 10.1136/bmjopen-2024-098505 (PMC12598997; doi:10.1136/bmjopen-2024-098505)
Supplement: online supplemental file 1 [file bmjopen-15-11-s001.docx]

**Supplemental Table 1: Federal member states, districts, and target population for the study**

| **State** | **Region** | **District** | **Target Under-Five Population** | | **Drought-Affected Population** | **Total Population** |
| --- | --- | --- | --- | --- | --- | --- |
|  |  |  | **Girls** | **Boys** |  |  |
| Puntland | Mudug | Galkaio | 18714 | 17480 | 208 936 | 497 531 |
| Puntland | Bari | Bossaso | 24291 | 22694 | 109 083 | 645 928 |
| Galmudug | Galgadud | Abudwak | 2896 | 2706 | 71 769 | 77 007 |
| Galmudug | Galgadud | Dhusamareb | 3481 | 3252 | 84 820 | 92 566 |
| Jubbaland | Gedo | Dolow | 3209 | 2998 | 29 400 | 85 335 |
| Southwest | Bay | Baidoa | 19870 | 19870 | 229 223 | 642 749 |
| Jubbaland | Lower Juba | Kismayo | 11745 | 10973 | 49 480 | 303 700 |
| Hirshabelle | Hiran | Baladweyne | 10814 | 10103 | 43 908 | 287 565 |
| Benadir | Benadir | Wadajir | 6722 | 6280 | 703 | 178 751 |
| **Total** | | | **101,742** | **96,356** | **827,322** | **2,811,132** |

**Supplemental Table 2. Logistic regression analysis to explore sociodemographic determinants of overall childhood morbidities and morbidities from pneumonia and diarrhea in Somalia.**

| Socio-economic factors | Any under-5 morbidity within a household in the last 3 months (N=2712) | | | Any under-5 pneumonia case within a household in the last 3 months (N=2712) | | | Any under-5 diarrhea case within the household in the last 3 months (N=2712) | | |
| --- | --- | --- | --- | --- | --- | --- | --- | --- | --- |
|  | Prevalence (71.0%) | Crude Odds Ratio (COR) with 95% CI | Adjusted Odds Ratio (AOR) with 95% CI | Prevalence (47.4%) | Crude Odds Ratio (COR) with 95% CI | Adjusted Odds Ratio (AOR) with 95% CI | Prevalence (40.3%) | Crude Odds Ratio (COR) with 95% CI | Adjusted Odds Ratio (AOR) with 95% CI |
| Asset quintile  Poorest  2^nd^  3^rd^  4^th^  Richest | 69.4%  79.7%  70.5%  77.4%  58.2% | Significance  (p) = <0.001  1.00  1.74 (1.32-2.30)  1.05 (.81-1.37)  1.51 (1.15-1.97)  0.61 (.48-0.77) | Significance  (p) = <0.001  1.00  1.35 (.98-1.85)  0.80 (.59-1.10)  0.62 (.43-.90)  0.30 (.20-0.45) | 54.5%  63.5%  58.9%  68.8%  30.3% | Significance  (p) =<0 .001  1.00  1.48 (1.16-1.89)  1.22 (0.96-1.55)  1.57 (1.23-2.00)  0.37 (0.29-0.47) | Significance  (p) = <0.001  1.00  1.35 (1.03-1.78)  1.06 (0.81-1.41)  0.88 (0.63-1.22)  0.52 (0.36-0.75) | 45.2%  45.1%  47.3%  36.8%  24.4% | Significance  (p) = .001  1.00  0.99 (0.75-1.31)  1.09 (0.82-1.44)  0.71 (0.53-0.94)  0.39 (0.28-0.54) | Significance  (p) = 0.01  1.00  1.28 (0.95-1.72)  1.24 (0.90-1.71)  0.87 (0.60-1.26)  0.67 (0.40-1.13) |
| Maternal education  Illiterate  Islamic  primary Incomplete  secondary  Secondary & above | 87.8%  63.6%  72.7%  80.8%  56.4% | Significance  (p) = <0.001  1.00  0.24 (.19-.32)  0.37 (.27-.51)  0.58 (.35-.97)  0.18 (.12-.26) | Significance  (p)= <0.001  1.00  0.27 (.20-.36)  0.36 (0.25-.51)  0.89 (.51-1.55)  0.51 (.32-.80) | 67.1%  51.5%  54.1%  49.6%  34.4% | Significance  (p)=<0.001  1.00  0.52 (.42-.63)  0.58 (.45-.75)  0.48 (.33-.71)  0.26 (.18 -.36) | Significance  (p) = <0.001  1.00  0.44 (0.36-0.55)  0.58 (0.43-0.78)  0.86 (0.55-1.34)  0.49 (0.33-0.75) | 39.8%  45.7%  34.6%  27.2%  28.8% | Significance  (p) =<0.001  1.00  1.27 (1.20-1.57)  0.80 (0.60-1.07)  0.56 (0.35-0.90)  0.61 (0.39-0.95) | Significance  (p) = 0.17  1.00  1.24 (0.97-1.59)  0.93 (0.67-1.28)  0.86 (0.52-1.43)  1.01 (0.60-1.70) |
| Maternal Occupation  Housewife  Day-labour  Business  Service | 72.9%  75.1%  67.8%  46.6% | Significance  (p) =<0.001  1.00  1.12 (.81-1.56)  .78 (.57-1.07)  .32 (.24-.44) | Significance  (p) = 0.002  1.00  0.74 (0.50-1.09)  1.02 (0.70-1.49)  0.50 (0.33-0.74) | 57.1%  48.3%  49.2%  32.8% | Significance  (p) =<0.001  1.00  0.70 (0.53-0.94)  0.73 (0.55-0.98)  0.37 (0.26-.051) | Significance  (p) = 0.002  1.00  0.79 (0.57-1.11)  1.09 (0.77-1.54)  0.49 (0.33-0.74) | 42.3%  39.4%  27.7%  25.5% | Significance  (p) =<0.001  1.00  0.89 (0.63-1.24)  0.52 (0.36-0.77)  0.46 (0.28-0.77) | Significance  (p) = 0.84  1.00  0.88 (0.61-1.27)  0.70 (0.47-1.06)  0.55 (0.31-0.99) |
| Area of residence  IDP  Rural  Urban | 63.1%  94.5%  70.3% | Significance  (p) = <0.001  1.00  10.12 (5.15-19.9)  1.38 (1.09-1.76) | Significance  (p)= 0.211  1.00  0.33 (0.03-3.84)  1.30 (0.91-1.85) | 66.7%  55.1%  42.3% | Significance  (p)=<0.001`  1.00  2.73 (1.88-3.98)  1.66 (1.33-2.11) | Significance  (p)= 0.009  1.00  0.06 (0.01-0.15)  1.69 (1.21-2.35) | 53.1%  36.4%  39.0% | Significance  (p)=0.005  1.00  0.51 (034-0.76)  0.57 (0.43-0.76) | Significance  (p)= 0.017  1.00  0.53 (0.34-0.83)  0.70 (0.51-0.96) |
| Region  Banadir  Bari  Bay  Galgadud  Gedo  Hiran  Lower Jubba  Mudug | 55.1%  62.8%  72.1%  99.4%  56.7%  95.6  65.0%  86.1% | Significance  (p) = <0.001  1.00  1.38 (1.05-1.81)  2.11 (1.64-2.71)  146.4 (36.12- 593.7)  1.07 (0.82-1.40)  17.54 (8.47-6.34)  1.52 (1.07-2.15)  5.06 (3.21-7.97) | Significance  (p)= <0.001  1.00  2.00 (1.39-2.87)  1.83 (1.38-2.42)  204.0 (49.8-835.5)  1.40 (0.97-2.04)  30.88 (2.42-393.6)  2.14 (1.42-3.22)  11.68 (6.89-19.81) | 47.5%  39.6%  61.6%  90.9%  48.9%  67.8%  37.2%  23.2% | Significance  (p) = <0.001  1.00  0.73 (0.56-0.96)  1.77 (1.39-2.25)  11.00 (7.41-16.3)  1.06 (0.81-1.38)  2.33 (1.63-3.32)  0.66 (0.47-0.93  0.34 0.23-0.49) | Significance  (p)= <0.001  1.00  1.06 (0.75-1.48)  1.65 (1.27-2.14)  12.84 (8.44-19.52)  1.72 (1.20-2.46)  2.58 (1.06-65.14)  0.86 (0.57-1.27)  0.53 (0.36-0.82) | 40.3%  39.1%  48.9%  40.1%  49.0%  36.0%  27.7%  24.1% | Significance  (p)=<001  1.00  0.95 (0.67-1.35)  1.41 (1.05-1.91)  0.99 (0.73-1.35)  1.41 (1.00-2.03)  0.83 (0.57-1.23)  0.57 (0.36-0.90)  0.47 (0.31-0.72) | Significance  (p)= 0.30  1.00  1.20 (0.76-1.89)  1.41 (1.03-1.94)  1.13 (0.80-1.61)  1.12 (0.72-1.76)  0.48 (0.18-0.84)  0.60 (0.36-0.99)  0.81 (0.46-1.43) |
| Cooking materials  Gas and Electricity  Charcoal and Wood | 50.6%  75.1% | Significance  (p) = <0.001  1.00  2.94 (2.40-3.60) | Significance  (p) = <0.001  1.00  1.82 (1.32-2.51) | 30.0%  59.1% | Significance  (p) = <0.001  1.00  3.37 (2.71-4.19) | Significance  (p) = <0.001  1.00  1.77 (1.30-2.39) | 24.9%  42.4% | Significance  (p) = <0.001  1.00  2.22 (1.63-3.04) | Significance  (p) = 0.089  1.00  1.43 (0.95-2.17) |
| Type of latrine  Sanitary  Unsanitary | 80.5%  19.5% | Significance  (p) = 0.26  1.00  0.68 (0.35-1.34) | Significance  (p) = 0.12  1.00  0.50 (0.21-1.20) | 53.7%  56.6% | Significance  (p)= 0.23  1.00  1.12 (0.93-1.36) | Significance  (p)= 0.78  1.00  1.05 (0.76-1.45) | 38.3%  48.7% | Significance  (p)= <0.001  1.00  1.53 (1.22-1.92) | Significance  (p)= 0.52  1.00  1.12 (0.79-1.59) |
| Source of Drinking Water  Safe drinking water  Unsafe drinking water | 71.3%  65.0% | Significance  (p) = 0.11  1.00  0.75 (0.52-1.07) | Significance  (p) = 0.012  1.00  0.57 (0.37-0.88) | 55.1%  39.3% | Significance  (p) = <0.001  1.00  0.53 (0.37-0.75) | Significance  (p) = 0.004  1.00  0.57 (0.38-0.84) | 40.1%  45.2% | Significance  (p) =0.33  1.00  1.23 (0.81-1.87) | Significance  (p) = 0.66  1.00  0.92 (0.57-1.47) |

**Supplemental Table 3. Logistic regression analysis to explore sociodemographic determinants of access to care (pneumonia, diarrhea, and delivery care in Somalia)**

| **Socio-economic factors** | **Accessed to health facility for treatment of pneumonia by U5 children (N=1472)** | | | **Accessed to health facility for treatment of diarrhea by U5 children (N=783)** | | | **Accessed to facility for delivery of the last child**  **(N=2712)** | | |
| --- | --- | --- | --- | --- | --- | --- | --- | --- | --- |
|  | **Percent accessed (53.5%)** | **Crude Odds Ratio (OR) with 95% CI** | **Adjusted Odds Ratio (OR) with 95% CI** | **Percent accessed (84.9%)** | **Crude Odds Ratio (OR) with 95% CI** | **Adjusted Odds Ratio (OR) with 95% CI** | **Percent accessed (62.2%)** | **Crude Odds Ratio (OR) with 95% CI** | **Adjusted Odds Ratio (OR) with 95% CI** |
| **Asset quintile**  Poorest  2^nd^  3^rd^  4^th^  Richest | 54.3%  53.0%  51.1%  52.3%  60.6% | Significance (p)=0.36  1.00  0.95 (0.70-1.30)  0.88 (0.64-1.21)  0.92 (0.68-1.26)  1.30 (0.88-1.90) | Significance  (p)= 0.19  1.00  0.97 (0.69-1.35)  0.91 (0.64-1.29)  0.96 (0.64-1.45)  1.67 (0.94-2.96) | 83.0%  90.4%  86.7%  78.6%  83.5% | Significance  (p)= 0.037  1.00  1.92 (1.04-3.57)  1.34 (0.74-2.40)  0.75 (0.43-1.30)  1.04 (0.51-2.12) | Significance  (p)= 0.075  1.00  2.71 (1.38-5.31)  1.58 (0.81-3.10)  1.58 (0.72-3.46)  1.95 (0.58-6.54) | 53.7%  51.7%  63.3%  71.3%  71.2% | Significance (p)=<0.001  1.00  0.93 (0.73-1.17)  1.49 (1.17-1.90)  2.14 (1.67-2.76)  2.13 (1.66-2.74) | Significance (p)=<0.001  1.00  1.38 (1.04-1.63)  1.94 (1.45-2.60)  2.53 (1.80-3.56)  4.25 (2.80-6.46) |
| **Maternal education**  Illiterate  Islamic  Primary  Incomplete Secondary  Secondary & above | 52.9%  51.2%  58.3%  62.9%  58.2% | Significance (p)=0.175  1.00  0.93 (0.73-1.19)  1.24 (0.90-1.73)  1.51 (0.87-2.61)  1.24 (0.74-2.09) | Significance  (p)= 0.17  1.00  0.86 (0.65-1.14)  1.23 (0.86-1.78)  1.38 (0.76-2.49)  0.95 (0.52-1.74) | 89.0%  81.0%  92.2%  82.1%  84.4% | Significance  (p)= 0.020  1.00  0.53 (0.32-0.85)  1.45 (0.63-3.35)  0.57 (0.20-1.63)  0.67 (0.24-1.89) | Significance  (p)= 0.014  1.00  0.55 (0.31-0.97)  2.03 (0.83-4.98)  0.80 (0.24-2.60)  0.89 (0.24-3.31) | 42.9%  65.0%  67.0%  76.8%  88.7% | Significance (p)=<0.001  1.00  2.47 (2.04-2.94  2.70 (2.08-3.50)  4.41 (1.83-6.87)  10.47 (6.54-16.75) | Significance  (p)=<0.001  1.00  1.64 (1.31-2.05)  1.96 (1.45-2.64)  3.68 (2.22-6.11)  3.62 (2.11-6.20) |
| **Maternal Occupation**  Housewife  Day-labor  Business  Service | 53.8%  51.5%  48.0%  61.4% | Significance  (p) =0.421  1.00  0.91 (0.61-1.38)  0.79 (0.53-1.97)  1.37 (0.79-2.36) | Significance  (p) = 0.27  1.00  1.07 (0.69-1.67)  0.65 (0.42-1.02)  1.10 (0.59-2.04) | 85.2%  85.2%  78.9%  85.7% | Significance  (p) =  1.00  1.00 (0.48-2.10)  0.65 (0.29-1.46)  1.04 (0.30-3.60) | Significance  (p) = 0.861  1.00  0.78 (0.34-1.80)  0.73 (0.29-1.83)  1.11 (0.27-4.64) | 59.7%  53.7%  70.9%  93.1% | Significance  (p) = <0.001  1.00  0.78 ()  1.64 ()  5.03 () | Significance  (p) = 0.07  1.00  1.05 (0.75-1.47)  1.07 (0.74-1.55)  2.42 (1.25-4.68) |
| **Area of residence**  IDP  Rural  Urban | 51.4%  46.7%  54.5% | Significance  (p) = <0.231  1.00  0.83 (0.51-1.35)  1.13 (0.80-1.60) | Significance  (p)= 0.211  1.00  2.38 (2.24-2.48)  1.20 (0.75-1.92) | 92.0%  79.4%  84.2% | Significance  (p)=<0.001`  1.00  0.33 (0.13-0.83)  0.46 (0.23-0.94) | Significance  (p)= 0.011  1.00  1.17 (0.65-2.09)  0.24 (0.09-0.61) | 73.5%  31.7%  63.1% | Significance  (p)=<0.001  1.00  0.17 (0.11-0.25)  0.62 (0.48-0.80) | Significance  (p)= 0.974  1.00  1.15 (0.08-15.96)  0.96 (0.65-1.43) |
| **Region**  Banadir  Bari  Bay  Galgadud  Gedo  Hiran  Lower Jubba  Mudug | 56.1%  55.2%  53.5%  52.1%  57.7%  46.7%  55.2%  42.9% | Significance  (p) = 0.47  1.00  0.97 (0.64-1.46)  0.90 (0.65-1.24)  0.85 (0.62-1.18)  1.07 (0.73-1.57)  0.69 (0.45-1.05)  0.97 (0.30-1.13)  0.59 (0.30-1.13) | Significance  (p)= <0.001  1.00  0.78 (0.47-1.31)  0.92 (0.66-1.30)  0.88 (0.60-1.27)  1.22 (0.75-1.98)  0.78 (0.41-1.49)  0.80 (0.44-1.46)  0.37 (0.18-0.80) | 95.9%  86.7%  87.0%  73.6%  88.1%  79.0%  75.8%  86.8% | Significance  (p)= <0.001  1.00  0.28 (0.09-0.81)  0.28 (0.10-0.76)  0.12 (0.05-0.31)  0.31 (0.11-0.92)  0.16 (0.05-0.47)  0.13 (0.04-0.44)  0.28 (0.08-1.02) | Significance  (p)= <0.001  1.00  0.13 (0.04-0.47)  0.38 (0.14-1.06)  0.14 (0.05-0.38)  0.23 (0.07-0.80)  0.03 (0.00-0.06)  0.08 (0.02-0.28)  0.24 (0.05-1.15) | 66.1%  80.3%  47.7%  66.5%  85.2%  31.1%  64.4%  32.8% | Significance  (p)=<001  1.00  2.09 (1.52-2.85)  0.47 (0.37-0.60)  1.02 (0.77-1.35)  2.95 (2.10-4.15)  0.23 (0.16-0.33)  0.93 (0.65-1.32)  0.25 (0.18-0.36) | Significance  (p)= 0.30  1.00  1.21 (0.80-1.81)  0.56 (0.43-0.74)  0.78 (0.57-1.07)  3.67 (2.35-5.70)  0.31 (0.02-4.36)  0.65 (0.43-0.98)  0.09 (0.06-0.15) |
