# Supplementary material for: Burden and sociodemographic determinants of pneumonia and diarrhoea among children younger than 5 years in Somalia: a community-based cross-sectional study
Source: BMJ Open. 2025 Nov 9;15(11):e098505. doi: 10.1136/bmjopen-2024-098505 (PMC12598997; doi:10.1136/bmjopen-2024-098505)
Supplement: online supplemental file 2 [file bmjopen-15-11-s002.docx]

**Baseline Community Survey for Every Breath Counts Project of the WHO**

**Burden and sociodemographic determinants of pneumonia and diarrhea among under-five children in Somalia: A community-based cross-sectional study**

***Instruction to the Enumerator:***

1. Select 3 villages (selection of villages by supervisor and state focal point) randomly from catchment areas of each MCH center. Thus, a total of 36 villages from around 12 MCH centers.
2. From each village 60 interviews will have to be conducted and thus a total of 2767 interviews will be conducted in Somalia.
3. First you go to the central place of the village and identify a household randomly to start the survey. If there is more than one under-5 child in a household, select one at random. If there are no under-5 children in the household leave the house and move to the next house.
4. The direction of movement will also have to be at random. Take a coin (or a currency note). If the last digit of the coin or note is 1 or 6, move to the North to select the next household. If the last digit is 2 or 7 move to the east, if 3 or 8 move to the south, if 4 or 9 move to the west. If the last digit is 5 or 0 take another coin (or note) to select direction of movement in a similar way. Continue interviews till 60 mothers (with under-5 children are covered).
5. Remember 60 completed interviews we need from each cluster. If 60 under-5 children are not covered from in one village move to the next village to cover the target.
6. Please don’t forget to GREET the respondent and always take written consent after explaining the purpose of the study.
7. If you face any problem, consult your supervisor or study investigators immediately.
8. The respondent will have to be a married woman of reproductive age (MWRA) with children under 5 years of age. If there is more than one such MRWA with under-5 children, select one randomly. If there are no such women [MWRA with under 5 children], go to the next household). Continue data collection till 60 interviews are completed from the cluster (village).

***Introduction and Consent Process:***

Hello. My name is __________________________________. I am working with the

NIH authority. WHO Somalia is implementing a project to improve health outcome of under-5 children suffering from pneumonia and diarrhoea through imparting a package of cost-effective and evidence-based community and facility level interventions in selected districts of the country. We are conducting this baseline community survey to benchmark the current morbidity, mortality and care seeking pattern for diarrhoea and pneumonia among under-5 children for future evaluation through a similar endline community survey. The information we collect will also help the government to plan future IMNCI services in the country. This household has been randomly selected for the survey. I would like to ask you some questions about your household. The survey usually takes about 40 to 45 minutes to complete all questions. All personnel involved in this survey will maintain strict confidentiality and anonymity of all information obtained during the interview that pertains to any household or individual. The data gathered will be released only in the form of statistical summaries in which no reference to any person shall appear. You don't have to be in the survey, but we hope you will agree to answer the questions since your views are important. In case you need more information about the survey, you may contact the ------------------ (phone no ---------------------------; email: ___________________).

Do you have any questions?

I give consent to take part in the survey.

Signature of the respondent _________________

Time: _____ ______ hours

Date: ___/____/______

***Questionnaire for Quantitative Baseline Community Survey***

1. **Socio-demographic characteristics**
2. Name of the respondent_____________________________
3. Age (in complete years) ____________years
4. Village _________________________________
5. Settlement Type: Urban/Rural/ Nomadic/IDP______________________
6. District____________________________
7. Region ____________________________
8. Para (No of births) ________
9. Gravida (Number of conception/pregnancy) __________
10. Number of household members _______ persons
11. Education of the respondent
12. No education
13. Islamic
14. Primary
15. Incomplete secondary
16. Secondary
17. Higher secondary and above
18. Occupation(respondent/women)
19. Housewife
20. Service (private/public)
21. Business
22. Day-labour
23. Others (Mention_)
24. Total monthly family income (__________________USD/month)
25. Type of latrine used by family members.
26. Sanitary
27. Poor Flush Water-seal latrine
28. Pit latrine
29. Open Space / Bush
30. Others (mention__)
31. Source of drinking water?
32. Piped water
33. Tubewell
34. Surface water
35. Others (mention___)
36. Cooking material
37. Electricity
38. Gas
39. Wood / leaves
40. Charcoal
41. Others (mention_)
42. Construction materials for main dwelling house (Observe and record)
43. Roof
44. Concrete
45. Tin (corrugated sheet)
46. Plastic sheets
47. Grass/leaves
48. Others (mention)
49. Wall
50. Concrete
51. Tin
52. Mud
53. Grass/leaves
54. Others___
55. Floor
56. Concrete
57. Wooden
58. Mud
59. Others__
60. Total number of bedrooms in the household? _________________
61. Do the household possess the followings? (Multiple response) (Tick)
62. Private car
63. Motorcycle
64. Bicycle
65. Freeze
66. Live stocks
67. Radio
68. TV
69. Air-conditioner
70. Ceiling Fan
71. Microwave
72. Computer / laptop /i-pad/ tab
73. Mobile phone
74. Any Firming lands? Yes/No
75. If yes, total farming land in hectares ___. ____ hectares
76. Wi-Fi connection
77. Mobile data package
78. Any bank accounts
79. Mobile account
80. **Birth history**
81. Total number of births in the household in last one year ______
82. Total number of live births in the household in last one year ______
83. Total number of stillbirths in the household in last one year ________
84. Place of last birth?
85. Home
86. MCH center
87. District Hospital
88. Private hospital
89. NGO Hospital
90. Others (Mention__)
91. Who conducted the last birth?
92. Family members
93. Neighbors
94. Relatives
95. TBA (Traditional Birth Attendant)
96. CHW (Community Health Workers)
97. Midwife / Nurse
98. Doctor
99. Obstetrician
100. Others (mention__)

23.1. Outcome of last delivery

1. Singleton live-birth
2. Twin (both live births)
3. Twin (one live birth and one stillbirth)
4. Singleton stillbirth
5. Twin (both still birth)

23.2. When did you start breastfeeding for the last baby born?

1. immediately after birth
2. within 24 hours
3. between day 1 and day 7
4. after 1^st^ week of birth
5. never

23.3. Any neonatal complication within 1st week of birth? a) yes b) No

23.4. If yes, which ones? (multiple choice)

a) prematurity b) birth asphyxia c) low birth weight

d) birth injury e) jaundice f) other infection (name__)

23.5. Any PNC (Post-natal Care) visit? a) yes b) no

23.6 If yes how many PNC (Post-natal care) visit made? __________________ visit

23.7 Any ANC (antenatal care) visit during last pregnancy? Yes/No

(if no, go to Q 24)

23.8. If yes, number of ANC visit made? _________visits

23.9 Who provided services during the last ANC visit?

a) CHW

b) Nurse/midwives

c) Doctor

d) Obstetrician

e) Others (mention__________________)

1. **Under 5 Children**
2. Total number of under-5 children in the household __________
3. Total number of infants (under I year) in the household ________
4. Any under-5 death in the household in last one year? Y/N

(if no skip to q31)

1. If yes, total no of under 5 deaths in last 1 year _____
2. Age of last under-5 death (in month). _______months­­­­­­_______days
3. Probable cause of last under-5 death?
4. ARI
5. Diarrhea
6. Measles
7. Undernutrition
8. TB
9. Malaria
10. Congenital disease
11. Accident/injury/drowning
12. Others (Mention_)
13. Don’t know
14. Total number of infant deaths in the household in last one year __________
15. **Under-5 Morbidity and Care seeking**
16. Did any under-5 children within the household become sick during the last 3 months?
17. Yes
18. No (Go to question no 60)
19. If yes, how many children became sick during the last 90 days withing this household? ______
20. How many of them suffered from ARI (cough, fever, stridor. difficulty in breathing or chest-indrawing)? ________
21. How many children under 5 suffered from diarrhoea / frequent loose stool during last 90 days? ______
22. How many of them suffered from other diseases / health conditions? _________
23. Who was the last victim of ARI within last 3 months (Name of the child________________)
24. Sex of last ARI victim
25. Male
26. female
27. Age of last ARI victim? ________months?
28. What were the presenting symptoms and signs (multiple response possible)
29. Cough
30. Fever
31. Difficulty in breathing
32. Chest indrawing
33. Stridor
34. Unconsciousness
35. Others (mention___)
36. What is your perception about severity of the disease?
37. No pneumonia (common cold?)
38. Pneumonia
39. Very severe disease / Severe Pneumonia
40. Did he/she receive any treatment outside the home?
41. Yes
42. No (Go to question no 49)
43. If yes, from where?
45. MCH
46. Government Hospital
47. Private Hospital
48. Pharmacy
49. Traditional healers
50. Village doctors
51. Others (Mention___)
52. If treated in any health facility, was s/he admitted in the In-patient Department (IPD)?

1=yes

2=no

1. Which of the following treatments did s/he receive? (Multiple response possible)
2. Antibiotics tablet/capsule (show strips)
3. Antibiotic injection
4. Oxygen
5. Nebulizer
6. Bronchodilator (Syrup/inhaler)
7. Steroid tablet
8. Others (Mention______)
9. How much distance did you cover to reach the health facility? ______Km
10. Time required to reach the facility. _______minutes
11. Total household cost for the treatment of ARI _______USD
12. What was the outcome of treatment?
14. Cured
15. Improved
16. Referred to higher level health facility
17. Died
18. Others _____
19. Did any under 5 child suffered from diarrhoea in last 90 days? Y/N

(if no go to Q60)

1. Who was the last victim of diarrhoea? Name______________
2. Sex of the last diarrhea victim?
3. Male
4. Female
5. How old was s/he during the diarrhoeal attack? _________ months
6. Did he/she receive any treatment for diarrhoea outside the home?
7. Yes
8. No (Go to question no 60)
9. If yes, from where?
   1. MCH
   2. Government Hospital
   3. Private Hospital
   4. Pharmacy
   5. Traditional healers
   6. Village doctors
   7. Others (Mention__)
10. Did he receive the following drugs? (Multiple response possible)
11. ORS
12. IV Fluid
13. Zinc tablet / syrup
14. Antibiotics
15. Others (Mention_______)
16. Outcome of the treatment?
    1. Cured
    2. Improved
    3. Died
    4. Others _____
17. The distance between home and the health facility visited for treatment of diarrhoea. _____Km
18. Time required to visit the health facility. _______________
19. Total cost for the treatment of Diarrhoea ______ USD (include all costs including cost for consultation, bed occupancy, medicine, food, transport, and attendant/accompanying person)
20. How was the behavior of the service provider during your last visit to the health facility?
21. Excellent
22. Good
23. Average
24. Bad
25. Very bad
26. Opinion about availability of medicine during last facility visit.

1= All prescribed medicines were available

2= Some of the prescribed medicines were available

3= None of the prescribed medicines were available

4= Don’t know / No comment

1. Opinion about overall quality of care in the health facility during your last facility visit
   1. Excellent
   2. Good
   3. Average
   4. Poor
   5. Very poor
2. Any suggestions to improve the quality and coverage of child-health care services? (Open ended, multiple response possible) __________________
3. Did any CHW/Marwo Caafimaad visit this household during the last 3 months?

1= Yes

2= No

1. If yes, how many visits did she make in the last 3 months? ______visits
2. In the last visit which services did she provide? (Multiple response possible)
3. Health education (BCC)
4. Iron and folic acid
5. Vitamin A capsule
6. Deworming tablet
7. Management / referral of diarrhoea
8. Management / referral of ARI/pneumonia
9. ANC (Antenatal care)
10. PNC
11. Screening for diabetes / hypertension
12. Growth monitoring
13. Risk communication
14. Other service (mention) __
