# Supplementary material for: Burden and sociodemographic determinants of pneumonia and diarrhoea among children younger than 5 years in Somalia: a community-based cross-sectional study
Source: BMJ Open. 2025 Nov 9;15(11):e098505. doi: 10.1136/bmjopen-2024-098505 (PMC12598997; doi:10.1136/bmjopen-2024-098505)
Supplement: Supplementary file 3 [file bmjopen-2024-098505.reviewer_comments.pdf]

PEER REVIEW HISTORY

BMJ Open publishes all reviews undertaken for accepted manuscripts. Reviewers are asked to complete a checklist review form and are provided with free text boxes to elaborate on their assessment. These free text comments are reproduced below.

ARTICLE DETAILS

Title (Provisional)

Burden and sociodemographic determinants of pneumonia and diarrhea among children younger than 5 years in Somalia: a community-based cross-sectional study

Authors

Tahlil, Abdullahi Ahmed; Osman, Mariam Muse; Gedi, Saido; Haji, Fartun Ali; Mohamed , Abdulmunim; Asir, Abdirashid; Al Azad, Abdullahi; Anwar, Iqbal; Ahmed, Abdirahman Abdirizak; Abdulle, Yahye Sheikh; Garba, Bashiru; Mohamed, Mohamed Abdelrahman

---

VERSION 1 - REVIEW

**Reviewer**	**1**
**Name**	**Bilchut, Awraris**
**Affiliation**	**Debre Berhan University, Public health**
**Date**	**15-Mar-2025**
**COI**	**None**

Question for the authors

• What contributions does the research add to the existing knowledge?

• Could it be possible to clearly indicate what the research question is. The objective is vague and not clear.

• Do you believe the presenting style and population overview are sufficient and illustrative?

• Can we assess the quality of care by simply asking good, very good, or excellent?

General comments

Scientific reliability:

• The research question is not adequately stated or addressed.

• The presenting style and population overview are insufficient and illustrative

Methods:

• The objective is unclear and not explicit enough to measure, thus it is still unclear.

• Difficult to look the main outcome and its clear measurement.

Results:

• As the objective is not clear the result and discussion lack clarity

• Sociodemographic could be good if presented by number and percentage

• The way behavior measured and quality of service assessed is not clear and needs clear justification in the method part

• The way of assessment of the quality of care is not clear

References:

• The way the references presented is not correct and some of them are outdated.

Supplement documents:

• Figures and tables are excellent and explanatory

**Reviewer**	**2**
**Name**	**Sialubanje, Cephas**
**Affiliation**	**Ministry of Health, Levy Mwanawasa Medical University**
**Date**	**02-May-2025**
**COI**	**None**

The manuscript is well written. However, it needs some revision in the methods, results and discussion sections before it can be considered for publication

Methods: The authors mention that they conducted a survey and collected data using a semi-structured questionnaire. But they also mention that they conducted interviews. The two are different. A questionnaire is administered. Let them clarify.

Results: The results are not well presented and quite confusing, especially table 6 on logistic regression. Let the authors separate the findings based on the outcome of interest

Discussion: The discussion needs to be written succinctly with the first paragraph summarising the aim of the study and major findings. The subsequent paragraphs should discuss and corroborate the findings; not repeating the methods. The study limitations are not clearly presented and discussed.

**Reviewer**	**3**
**Name**	**Kelson, Zoe**
**Affiliation**	**University of Exeter, Mathematics**
**Date**	**09-Jun-2025**
**COI**	**None**

This study aims to understand the major determinants of pneumonia and diarrhea among under five year-old children in Somalia.

Reviewer comments:

"Community-based survey was conducted using modified WHO EPI Cluster sampling technique to identify households and respondents in the 9 selected districts across six member states in Somalia" [Abstract]

and

"A community-based cross-sectional study was conducted over a period of 18 months in 9 districts of Somalia (Table 1)... The study was conducted in 12 PHC/MCH clinics which act as referral hospitals for the 9 districts... modified WHO EPI Cluster sampling technique to identify households and respondents in the community to conduct interviews"

Can the authors please provide details on the sampling weights that should be applied to adjust for the survey design?

"Taking 1.5 as design-effect as we deviated from simple random sampling (SRS) we needed 1785*1.5 = 2678 households to visit. It implies that following EPI 36 cluster sampling methodology, respondents needed to visit at least 75 households in a village to interview mothers of under-5 children.

Table 2. Sample size calculation "

The authors have suitably communicated the basis for the sample size calculation.

"A semi-structured interviewer administered questionnaire was developed for data collection with closed, semi-closed and open-ended questions to address the study objectives."

Can the authors please acknowledge the potential for self-reporting and recall bias?

"After end of everyday data collection, the data were transferred to EXCEL and shared with all supervisors and study investigators to review meticulously for any inconsistencies in data

and necessary feedback given to the data collectors to rectify the data, if necessary, through repeating the interview revisiting the household where data anomaly was found."

Can the authors please confirm if there was any missing data?

"The logistic regression model was employed to examine the determinants of childhood pneumonia and diarrhea" [Abstract]

and

"For all data analysis including multivariate logistic regression analysis, we used SPSS-24 software...

An inequity analysis was undertaken to examine the variation in outcome indicators by socio-demographic makers along with logistic regression analysis to look for sociodemographic determinants of the study outcome indicators. A summary 'asset quintile' was developed combining asset variables in the survey through statistical methods of Principal Component and Factor Analysis (PCA & FA). "

Appropriate modelling methods have been applied.

Can more detail on the models please be provided here (such as listing the covariates included in the multivariate models, as well as specifying the outcome variables for instance)?

"A total of 2712 households were covered from 36 villages from the catchment areas of the 12 MCH centers. "

and

"Table 3. Sociodemographic characteristics of respondents "

and

"In 2712 households surveyed, a total of 19,660 individuals were residing of which 5,417 were of under-five age (27.6%)."

Can the authors please comment on whether they consider the included sample to be representative, for generalisability of the study findings?

"Our calculations suggest that the prevalence of overall under-5 morbidity was 468 cases per 1000 U5 children in the last 90 days. While the prevalence of pneumonia and diarrhea were 316 and 152 cases per 1000 U5 children in the last 90 days respectively... Our exploration depicts an under-5 mortality rate of 39.3 deaths per 1000 livebirths per year and an infant mortality rate of 20.8 per 1000 live births per year in the study area which is much lower than earlier estimates. The crude birth rate in the study area is 106 births per 1000 population while the stillbirth rate is found to be 149.8 per 1000 births" [Abstract]

and

"During the last year, a total of 70 under-5 deaths took place of which 37 deaths took place at age below one year (infants). Our exploration depicts an under-5 mortality rate of 39.3 deaths per 1000 livebirths per year and an infant mortality rate of 20.8 per 1000 live births per year in the study area which is much lower than earlier estimates. The crude birth rate in the study area is 106 births per 1000 population while the stillbirth rate is found to be 149.8 per 1000 births, which is very high. (Table 4) "

and

"Our calculations suggest that the prevalence of overall under-5 morbidity was 458 cases per 1000 U5 children in the last 90 days. While the prevalence of pneumonia and diarrhea were 316 and 152 cases per 1000 U5 children in the last 90 days respectively. In another way 63.1% families had at least one pneumonia case and 30.4% had at least one diarrhea case within last 90 days. The burden of childhood pneumonia was reported to be much higher than that of diarrhea but comparable with earlier estimates. (Table 5)"

Can uncertainty please be quantified and communicated for all estimates reported in this study?

"Children from the richest quartile household were 48% less likely to have pneumonia (OR 0.52, CI 0.36 to 0.75) and children of mothers with secondary and above education were 51% less likely to have pneumonia in last 3 months (OR 0.49, CI 0.33 to 0.75)."

and

"the richest quartile household was 4.25 times more likely to be delivered in a health facility than a child from the poorest quartile (OR 4.25, CI 2.80 to 6.46). Children of mothers with secondary and above education were 3.62 times more likely to be delivered in a health facility than children of housewives (OR 3.62, CI 2.11 to 6.20)"

and

"a child from wealthy household was 70% less likely to have any under-5 morbidity (OR 0.30, CI 0.20 to 0.45) compared to his counterpart from a poor family"

Can the authors please more accurately infer statistically significant differences in odds ratios (i.e. revising the terms 'less likely' and 'more likely') throughout the Results and Discussion?

"Children belonging to the richest quintile were 43% less vulnerable to diarrhea (OR 0.67, CI 0.40 to 1.13) compared to the poorest quartile."

Can the authors please revisit the interpretation of this OR, which crosses 0?

"children from richest quintile household were 1.67 times (OR 1.67, CI 0.96 to 2.94) more likely to visit health facility than a child from poorest quintile household."

Similarly, can the interpretation of this statistical outcome please be amended?

"This study utilizes a cross-sectional design, which may limit the ability to capture temporal associations or seasonal variations in the incidence of pneumonia and diarrhoea among

under-five children, particularly given the higher prevalence of these illnesses during the wet season. • This study did not assess breastfeeding practices as a potential sociodemographic determinant, which might have provided valuable insights into the relationship between infant feeding practices and the prevalence of pneumonia and diarrhoea in the target population." [strengths and limitations of this study]

Can the Discussion of the study limitations please be expanded on?

Thanks for providing a copy of the STROBE checklist.

VERSION 1 - AUTHOR RESPONSE

Reviewer 1	
**Reviewer's Comment**	**Author's Response**
**Question for the authors** What contributions does the research add to the existing knowledge?	We appreciate your recognition of our efforts. In the revised manuscript, we have explicitly highlighted our study's unique contributions. These additions clarify how our research advances current understanding in this field.
Could it be possible to indicate what the research question is? The objective is vague and not clear.	We appreciate this observation. We have revised the objective to clearly state the research question in the manuscript as follows: "This study aims to determine the burden (prevalence) and identify key socio-demographic and environmental determinants of pneumonia and diarrhea among under-five children in Somalia." This reflects our intention to assess both disease prevalence and associated factors.
Do you believe the presenting style and population overview are sufficient and illustrative?	Thank you for the feedback. We believe the presentation is sufficient as it includes a comprehensive description of the study design, sampling, population characteristics (Table 3), regional distribution, and detailed statistical analysis (Tables 6-7). However, we remain open to specific suggestions to enhance clarity or visualization if required.
Can we assess the quality of care by simply asking good, very good, or excellent?	Thank you for raising this important methodological point. While assessing quality of care using subjective categories such as good, very good, and excellent has limitations, these perception-based assessments are widely used in humanitarian health surveys where objective measurement is not feasible due to resource and access constraints. In our study, we complemented perceived quality with objective indicators such as availability of medicines, treatment provided, distance, cost, and provider behavior to strengthen the validity of our findings on quality of care.
**General comments** **Scientific reliability:** The research question is not adequately stated or addressed.	We appreciate the reviewer's comment and would like to clarify that the research question is explicitly stated in both the Abstract and Introduction sections. The objective of the study was "to understand the major determinants of pneumonia and diarrhea among under-five children in Somalia" (Abstract, line 13; Introduction, line 78). Additionally, the methods, results, and discussion comprehensively addressed this question by exploring prevalence, determinants using multivariate logistic regression, and sociodemographic predictors influencing childhood pneumonia and diarrhea in a fragile humanitarian setting. This research question guided the design and analysis, ensuring scientific reliability throughout the study.
The presenting style and population overview are insufficient and illustrative.	Thank you for this feedback. We respectfully disagree that the presentation is insufficient, as we provided a comprehensive overview of the study population in the Methods section (Study Design and Setting,

	Table 1) and detailed sociodemographic characteristics in the Results section (Table 3). Additionally, we included detailed sampling techniques, sample size calculations, and community demographics, including household size, income, education, occupation, settlement type, and housing conditions.
**Methods:**<br>The objective is unclear and not explicit enough to measure; thus, it is still unclear.	We appreciate your comment. We have revised the objective to make it clearer and measurable. The revised objective now reads: "To estimate the prevalence and identify the socio-demographic and environmental determinants of pneumonia and diarrhea among under-five children in Somalia." This reflects the descriptive and analytical components of our study as presented in the methods and results sections.
Difficult to look at the main outcome and its clear measurement.	Thank you for this important feedback. In response, we have clarified in the methodology that our main outcomes were (1) prevalence of pneumonia and diarrhea (measured as self-reported probable cases by caregivers within the last 90 days using structured questionnaires), and (2) associated socio-demographic determinants, analysed using multivariate logistic regression models. We ensured that these outcome measurements are consistently described under "Data management and analysis" and reported in Tables 5 and 6 of the results sections to enhance clarity.
**Results:**<br>As the objective is not clear, the results and discussion lack clarity	We appreciate this observation. The objective of the study is stated under the abstract and introduction as "To understand the major determinants of pneumonia and diarrhea among under-five children in Somalia." The results and discussion were structured to address this objective by presenting the burden, prevalence, and sociodemographic determinants of these diseases. However, to improve clarity, we will rephrase the objective in the abstract and introduction to explicitly state: "This study aimed to determine the prevalence, burden, and associated sociodemographic determinants of pneumonia and diarrhea among under-five children in Somalia." This will ensure a direct link to the results and discussion.
Sociodemographics could be good if presented by number and percentage.	We appreciate your suggestion. In our manuscript, sociodemographic characteristics were indeed presented with both numbers and percentages, as shown in Table 3 (page 8 of manuscript), which shows detailed counts and proportions for variables such as education, occupation, settlement type, and construction materials. No further action required in terms of adding new data presentation.
The way behavior is measured and the quality of service assessed is not clear and needs clear justification in the method part.	We appreciate this comment. In the methods section under data collection tools, we used a structured questionnaire where respondents were asked to rate the behavior of service providers as "excellent," "good," "satisfactory," "bad," or "very bad," and quality of care as "excellent," "good," "satisfactory," or "poor," based on their most recent visit. This approach was adapted from standard WHO client satisfaction assessment tools. We have included a sentence in the methods part to clarify the measurement: *"Behavior and quality of service were assessed using client-reported Likert-scale items adapted from WHO standard patient satisfaction survey modules."*
The way of assessing the quality of care is not clear.	Thank you for raising this important point. Quality of care was assessed based on user perception, using questions regarding provider behavior, availability of medicines, and overall satisfaction with care received, rated on a Likert scale as mentioned above. In addition, respondents were asked about specific treatments received (e.g., antibiotics, ORS, zinc) to assess clinical quality.

**References:** The way the references are presented is not correct, and some of them are outdated.	Thanks for the comments. We have updated several references with the most recent and relevant literature to ensure that the study's context is current and credible.
**Supplement documents:** Figures and tables are excellent and explanatory	We thank you for this positive feedback. We appreciate that the clarity of our figures and tables meets the required standard and helps to illustrate the results and sociodemographic determinants effectively. We have retained the tables and figures as they add substantial value to the manuscript's interpretation.

Reviewer 2	
**Reviewer's Comment**	**Author's Response**
**Methods:** The authors mention that they conducted a survey and collected data using a semi-structured questionnaire. But they also mention that they conducted interviews. The two are different. A questionnaire is administered. Let them clarify.	We appreciate the reviewer's comment and agree that this could be clearer. We clarify that the data were collected using a semi-structured questionnaire administered through face-to-face interviews conducted by trained enumerators. We have revised the Methods section and Abstract accordingly to reflect this, ensuring consistent terminology throughout the manuscript.
**Results:** The results are not well presented and quite confusing, especially table 6 on logistic regression. Let the authors separate the findings based on the outcome of interest.	We thank the reviewer for this important observation. We respectfully clarify that in our study, the logistic regression analysis in Table 6 was structured to display the association of multiple sociodemographic determinants with both pneumonia and diarrhea in a single model to allow direct comparison of risk factors across the two outcomes. This approach is consistent with our aim of identifying shared and distinct predictors within the same population.
**Discussion:** The discussion needs to be written succinctly, with the first paragraph summarizing the aim of the study and major findings. The subsequent paragraphs should discuss and corroborate the findings, not repeating the methods. The study limitations are not presented and discussed.	Thank you for your comment. We agree that the discussion should be more concise. We have revised the section to remove redundant information and enhance clarity while retaining key insights. The revised opening paragraph of the discussion succinctly states the study objective and key findings, setting the stage for the detailed discussion. We removed methodological repetition and focused on interpreting findings in light of existing literature. Finally, we have added a dedicated paragraph discussing the limitations, including the cross-sectional design, potential recall bias, and lack of data on breastfeeding practices.

Reviewer 3	
**Reviewer's Comment**	**Author's Response**
"Community-based survey was conducted using modified WHO EPI Cluster sampling technique to identify households and respondents in the 9 selected districts across six member states in Somalia" [Abstract] and "A community-based cross-sectional study was conducted over 18 months in 9 districts of Somalia (Table 1). The study was conducted in 12 PHC/MCH clinics, which act as referral hospitals for the 9 districts... modified WHO EPI Cluster sampling technique to	We thank the reviewer for this valuable and insightful comment. In response, we have clarified and expanded the "Data Management and Analysis" section of the manuscript to include a detailed explanation of the sampling weights used to adjust for the survey's cluster design. Although our modified WHO EPI 30-cluster sampling technique employed a fixed number of households (75) per village, we recognize that underlying differences in population size across clusters and districts could

identify households and respondents in the community to conduct interviews" Can the authors please provide details on the sampling weights that should be applied to adjust for the survey design?	influence the probability of household selection. Therefore, sampling weights were computed as the inverse of the probability of selection and incorporated into our descriptive and multivariate analyses to ensure that the findings are representative at the population level. These sampling weights accounted for: 1. The probability of selecting a cluster (village) within each district, and 2. The probability of selecting a household within the selected village. We also clarified in the revised text that the inclusion of these weights enhances the generalizability and validity of our estimates. The updated section explicitly describes how the weights were calculated and applied, and how they were used to adjust for potential design effects. We have added the following revised text in the "Data Management and Analysis" section of the manuscript to reflect this clarification.
"Taking 1.5 as design-effect, as we deviated from simple random sampling (SRS), we needed 1785*1.55 = 2767 households to visit. It implies that following the EPI 36 cluster sampling methodology, respondents needed to visit at least 75 households in a village to interview mothers of under-5 children. Table 2. Sample size calculation. The authors have suitably communicated the basis for the sample size calculation.	Thank you. We appreciate your recognition. The sample size was calculated based on key indicators, adjusted using a design effect of 1.55, consistent with deviation from simple random sampling due to the use of EPI 36-cluster sampling methodology.
"A semi-structured interviewer-administered questionnaire was developed for data collection with closed, semi-closed, and open-ended questions to address the study objectives." Can the authors please acknowledge the potential for self-reporting and recall bias?	We thank you for this valuable suggestion. We have added a statement acknowledging the possibility of recall and self-reporting bias in the "Limitations" section.
"After end of everyday data collection, the data were transferred to EXCEL and shared with all supervisors and study investigators to review meticulously for any inconsistencies in data and necessary feedback given to the data collectors to rectify the data, if necessary, through repeating the interview revisiting the household where data anomaly was found." Can the authors please confirm if there was any missing data?	Thank you for raising this point. During field data collection, data were reviewed daily for inconsistencies. Households with missing or invalid data were revisited for corrections. Therefore, minimal to no missing data remained after the quality control process.
"The logistic regression model was employed to examine the determinants of childhood pneumonia and diarrhea" [Abstract] and "For all data analysis, including multivariate logistic regression analysis, we used SPSS-24 software... An inequity analysis was undertaken to examine the variation in outcome indicators by socio-demographic markers, along with logistic regression analysis to look for sociodemographic determinants of the study outcome indicators. A summary 'asset quintile' was developed combining asset variables	Thanks for the insightful comment. We have now revised the manuscript to include detailed information on the outcome variables used in the multivariate logistic regression models (i.e., pneumonia, diarrhea, and overall childhood morbidity), as well as all sociodemographic covariates included (e.g., maternal education, household wealth, region, occupation, type of latrine, cooking material, water source, area of residence).

in the survey through statistical methods of Principal Component and Factor Analysis (PCA & FA). " Appropriate modelling methods have been applied. Can more details on the models please be provided here (such as listing the covariates included in the multivariate models, as well as specifying the outcome variables, for instance)?	
"A total of 2712 households were covered from 36 villages in the catchment areas of the 12 MCH centers. " And "Table 3. Sociodemographic characteristics of respondents. In 2712 households surveyed, a total of 19,660 individuals were residing, of which 5,417 were of age (27.6%)." Can the authors please comment on whether they consider the included sample to be representative of the generalizability of the study findings?	We appreciate your request for clarification. The study employed a modified WHO EPI 30-cluster sampling methodology across six federal member states and nine districts, targeting the catchment areas of 12 MCH centers. While not nationally representative, the sampling ensured geographic and sociodemographic diversity. We believe the findings are generalizable to similar conflict-affected and underserved populations in Somalia.
"Our calculations suggest that the prevalence of overall under-5 morbidity was 468 cases per 1000 U5 children in the last 90 days. While the prevalence of pneumonia and diarrhea were 316 and 152 cases per 1000 U5 children in the last 90 days, respectively... Our exploration depicts an under-5 mortality rate of 39.3 deaths per 1000 live births per year and an infant mortality rate of 20.8 per 1000 live births per year in the study area, which is much lower than earlier estimates. The crude birth rate in the study area is 106 births per 1000 population, while the stillbirth rate is found to be 149.8 per 1000 births" [Abstract]<br>During the last year, a total of 70 under-5 deaths took place, of which 37 deaths took place at age below one year (infants). Our exploration depicts an under-5 mortality rate of 39.3 deaths per 1000 live births per year and an infant mortality rate of 20.8 per 1000 live births per year in the study area, which is much lower than earlier estimates. The crude birth rate in the study area is 106 births per 1000 population, while the stillbirth rate is found to be 149.8 per 1000 births, which is very high. (Table 4) " and "Our calculations suggest that the prevalence of overall under-5 morbidity was 458 cases per 1000 U5 children in the last 90 days. While the prevalence of pneumonia and diarrhea was 316 and 152 cases per 1000 U5 children in the last 90 days, respectively. In another way, 63.1% families had at least one pneumonia case, and 30.4% had at least one diarrhea case within the last 90 days. The burden of childhood pneumonia was reported to be much higher than that of diarrhea, but comparable with earlier estimates. (Table 5)" Can uncertainty please be quantified and communicated for all estimates reported in this study?	We agreed with you. Confidence intervals (CIs) were calculated for all key point estimates wherever feasible based on the available data. These were incorporated for the morbidity, mortality, and stillbirth estimates to communicate statistical uncertainty.

"Children from the richest quartile household were 48% less likely to have pneumonia (OR 0.52, CI 0.36 to 0.75) and children of mothers with secondary and above education were 51% less likely to have pneumonia in last 3 months (OR 0.49, CI 0.33 to 0.75)." And "the richest quartile household was 4.25 times more likely to be delivered in a health facility than a child from the poorest quartile (OR 4.25, CI 2.80 to 6.46). Children of mothers with secondary and above education were 3.62 times more likely to be delivered in a health facility than children of housewives (OR 3.62, CI 2.11 to 6.20)" And "a child from wealthy household was 70% less likely to have any under-5 morbidity (OR 0.30, CI 0.20 to 0.45) compared to his counterpart from a poor family" Can the authors please more accurately infer statistically significant differences in odds ratios (i.e. revising the terms 'less likely' and 'more likely') throughout the Results and Discussion?	We thanked the reviewer and concurred with this recommendation. The manuscript was revised to ensure that inferences of statistical significance aligned strictly with the 95% confidence intervals and p-values reported.
"Children belonging to the richest quintile were 43% less vulnerable to diarrhea (OR 0.67, CI 0.40 to 1.13) compared to the poorest quartile." Can the authors please revisit the interpretation of this OR, which crosses 0?	We appreciate your observation. We agree that the 95% confidence interval includes 1.0, indicating the result is not statistically significant. We have revised the sentence for accurate interpretation.
"Children from richest quintile households were 1.67 times (OR 1.67, CI 0.96 to 2.94) more likely to visit a health facility than a child from a poorest quintile household."<br>Similarly, can the interpretation of this statistical outcome please be amended?	Thank you for this important point. We acknowledge the wide confidence interval crossing 1.0, indicating a lack of statistical significance. The interpretation has been revised accordingly.
"This study utilizes a cross-sectional design, which may limit the ability to capture temporal associations or seasonal variations in the incidence of pneumonia and diarrhoea among under-five children, particularly given the higher prevalence of these illnesses during the wet season. This study did not assess breastfeeding practices as a potential sociodemographic determinant, which might have provided valuable insights into the relationship between infant feeding practices and the prevalence of pneumonia and diarrhoea in the target population." [strengths and limitations of this study] Can the Discussion of the study limitations please be expanded on?	We thank the reviewer for this recommendation. We have expanded the limitations to include potential recall bias due to self-reported morbidity, limited generalizability due to sampling catchment MCH areas, inability to capture long-term seasonal trends due to single survey timing, and exclusion of nutritional variables such as breastfeeding practices and child malnutrition status, which may confound pneumonia and diarrhea outcomes.

VERSION 2 - REVIEW

**Reviewer**	**2**
**Name**	**Sialubanje, Cephas**

Affiliation **Ministry of Health, Levy Mwanawasa Medical University**

Date **20-Aug-2025**

COI

The authors have attended to most queries raised by the reviewers. However, some revision is needed before the manuscript is accepted for publication.

Abstract:

Participant: the authors mention that "The interviewer started interviewing from house number 1 and continued till 75 households were covered to.....". These were not interviews as the authors mention later in the document that an interviewer- administered questionnaire was used to collect the data. Please correct this

Design: Please don't' mention cross-sectional survey; all surveys are cross-sectional in design

Results: Line 50..." We explored probable causes.....". This is a cross-sectional design and causality can not be ascertained. The authors can use words like factors associated with or determinants....Please correct this error

Introduction:

Page 3 line 109: therefore this study aims to determine the burden (prevalence)....leave out burden and use prevalence; this is what the study investigated

Line 95 and 96: the citation is old (2019). Please cite the latest literature

Methods: Page 3 line 122: These 9 districts have been targeted...Should read, these 9 districts were targeted....

sampling: Page 4 line 133-138: Please explain how the villages were selected from the MCH catchment areas

Results: page 9 line 272: We explored probable causes: see comment above

---

Reviewer **3**

Name **Kelson, Zoe**

Affiliation **University of Exeter, Mathematics**

Date **12-Aug-2025**

COI

Thanks to the authors for their responses and revisions. The following reviewer requests are raised:

Author response:

"Therefore, minimal to no missing data remained after the quality control process."

and

Article text:

"As a result of this process, missing data were minimized to a negligible level, thereby improving the overall quality and completeness of the dataset. "

Reviewer request:

Can the authors please specify how much is 'a negligible level' here?

Author response:

"The manuscript was revised to ensure that inferences of statistical significance aligned strictly with the 95% confidence intervals and p-values reported"

Reviewer request:

The authors still infer statistically significant ORs using 'less likely' and 'more likely'.

Can they please use more accurate terminology for the interpretation of ORs, such as positive or negative associations, or increased or decreased odds?

Article text:

"Children belonging to the richest quintile were less likely to have diarrhea (AOR 0.67; 95% CI: 0.40–1.13) compared to the poorest quartile; however, this difference was not statistically significant"

and

"Children from the richest quintile households were more likely to visit health facilities for treatment than those from the poorest households (OR 1.67, CI 0.96 to 2.94); however, this difference was not statistically significant. "

Reviewer request:

Furthermore, statistically non-significant AORs (with CIs including 1) are still being inferred as an association (i.e. 'less likely' and 'more likely') despite not being evidenced in the statistical analysis. Can the authors please re-phrase these sections to clearly infer no evidence of an association in this study?

VERSION 2 - AUTHOR RESPONSE

Reviewer 3

Reviewer's Comment	Author's Response	Position
Thanks to the authors for their responses and revisions. The following reviewer requests are raised:<br><br>Author response:<br>"Therefore, minimal to no missing data remained after the quality control process."<br>and<br>Article text:<br>"As a result of this process, missing data were minimized to a negligible level, thereby improving the overall quality and completeness of the dataset. "<br>Reviewer request:<br>Can the authors please specify how much is 'a negligible level' here?	Thank you for your valuable feedback. In response to your request for clarification regarding the phrase "a negligible level," we specify that the amount of missing data after the quality control process was less than 1% of the total dataset.	Line 173-174
Author response:<br>"The manuscript was revised to ensure that inferences of statistical significance aligned strictly with the 95% confidence intervals and p-values reported"<br>Reviewer request:<br>The authors still infer statistically significant ORs using 'less likely' and 'more likely'.<br>Can they please use more accurate terminology for the interpretation of ORs, such as positive or negative associations, or increased or decreased odds?	Thank you for your valuable feedback. We appreciate your emphasis on precise terminology when interpreting odds ratios (ORs). In our revised manuscript, we have taken care to align our language with statistical best practices. Specifically, instead of using terms like "more likely" or "less likely," we now refer to "increased" or "decreased odds," where appropriate. This terminology more accurately reflects the nature of the ORs and avoids potential misinterpretation. We believe these adjustments enhance the clarity and scientific rigor of our manuscript.	Line 338-459
Article text:<br>"Children belonging to the richest quintile were less likely to have diarrhea (AOR 0.67; 95% CI: 0.40–1.13) compared to the poorest quartile; however, this difference was not statistically significant"<br>and<br>"Children from the richest quintile households were more likely to visit health facilities for treatment than those from the poorest households (OR 1.67, CI 0.96 to 2.94); however, this difference was not statistically significant. "<br>Reviewer request:<br>Furthermore, statistically non-significant AORs (with CIs including 1) are still being inferred as an association (i.e., 'less likely' and 'more	Thank you for your valuable feedback. We have revised the manuscript to clarify that the observed differences in odds ratios for both diarrhea prevalence and health facility visits between children from the richest and poorest households are not statistically significant. Specifically, we have rephrased these sections to state that there is no evidence of an association in this study, given that the confidence intervals include 1. We appreciate your guidance in ensuring accurate interpretation of our findings.	Line 374-376<br>Line 389-391

likely') despite not being evidenced in the statistical analysis. Can the authors please re-phrase these sections to clearly infer no evidence of an association in this study?		

**Reviewer 2**		
**Reviewer's Comment**	**Author's Response**	**Position**
The authors have attended to most queries raised by the reviewers. However, some revision is needed before the manuscript is accepted for publication.	Thank you for your constructive feedback. We have carefully addressed all the queries raised by the reviewers and made the necessary revisions to improve the manuscript. We appreciate your valuable suggestions and look forward to your further guidance.	Entire Manuscript
Abstract:<br>Participant: the authors mention that "The interviewer started interviewing from house number 1 and continued till 75 households were covered to....". These were not interviews as the authors mention later in the document that an interviewer-administered questionnaire was used to collect the data. Please correct this<br>Design: Please don't' mention cross-sectional survey; all surveys are cross-sectional in design<br>Results: Line 50..." We explored probable causes....". This is a cross-sectional design and causality cannot be ascertained. The authors can use words like factors associated with or determinants.... Please correct this error	Thank you for your valuable feedback. We have made the following revisions in response to your comments:<br>We have clarified the description of data collection by replacing "interviewed" with "data were collected through an interviewer-administered questionnaire" to accurately reflect the methodology, as per your suggestion.<br>We have removed the mention of "cross-sectional survey" to avoid redundancy, acknowledging that all surveys conducted are inherently cross-sectional.<br>While our study explores probable causes and associated factors, we have clarified in the manuscript that our findings pertain to probable causes rather than established causality. Specifically, we now state that we "explored probable causes of under-5 deaths," and have provided detailed percentages for each cause, emphasizing the descriptive nature of our findings without implying causality.	Line 26-30<br>Line 38-39<br>Line 50-53
Introduction:<br>Page 3 line 109: therefore, this study aims to determine the burden (prevalence).... leave out burden and use prevalence; this is what the study investigated<br>Line 95 and 96: the citation is old (2019). Please cite the latest literature	Thank you for your valuable feedback. We have revised the sentence to replace "burden" with "prevalence" as suggested, ensuring it accurately reflects the focus of our study. Regarding the citation, we acknowledge that it is from 2019; however, we have chosen to retain this reference to maintain consistency with the foundational literature supporting our study. We believe it remains relevant and appropriate for contextualizing our research, and we appreciate your understanding in this matter.	Line 95-97<br>Line 109-111
Methods: Page 3 line 122: These 9 districts have been targeted...Should read, these 9 districts were targeted....	Thank you for your valuable feedback. We have made the necessary correction in the manuscript, changing "these 9 districts have	Line 121

sampling: Page 4 line 133-138: Please explain how the villages were selected from the MCH catchment areas<br>Results: page 9 line 272: We explored probable causes: see comment above	been targeted" to "these 9 districts were targeted" as suggested.<br>While our study explores probable causes and associated factors, we have clarified in the manuscript that our findings pertain to probable causes rather than established causality. Specifically, we now state that we "explored probable causes of under-5 deaths," and have provided detailed percentages for each cause, emphasizing the descriptive nature of our findings without implying causality.	

VERSION 3 - REVIEW

Reviewer 2
Name Sialubanje, Cephas
Affiliation Ministry of Health, Levy Mwanawasa Medical University
Date 10-Oct-2025
COI

The authors have addressed my concerns

Reviewer 3
Name Kelson, Zoe
Affiliation University of Exeter, Mathematics
Date 25-Sep-2025
COI

Many thanks to the authors for responding to the latest round of reviewer comments, amending the reporting of the study (in particular, revising the inference of study outcomes) as requested.
